# Anatomy of a seafloor spreading event captured by in situ seismogeodesy

**DOI:** 10.1038/s41586-026-10785-0

**Published:** 2026-07-08

**Authors:** Jean-Yves Royer, Jean-Arthur Olive, Sara Bazin, Valérie Ballu, Anne Briais, Lise Retailleau, Pierre-Yves Raumer, Edgar Lenhof, OHA-GEODAMS Scientific party, J. Beesau, R. Daniel, D. Dausse, S. Furst, A. Gros-Martial, C. Guerin, E. Klein, D. Pacaud, C. Poitou, J. Tanrin, L. Testut

**Affiliations:** 1 Geo-Ocean, CNRS, University of Brest, Ifremer, UMR6538, Plouzané, France; 2 Laboratoire de Géologie, CNRS, Ecole Normale Supérieure, PSL University, UMR 8538, Paris, France; 3 Laboratoire Littoral Environnement et Sociétés, CNRS, University of La Rochelle, UMR7266, La Rochelle, France; 4 Institut de Physique du Globe de Paris, CNRS, Paris Cité University, UMR7154, Paris, France; 5 Lab-STICC, ENSTA, CNRS, University of Brest, UMR6285, Brest, France

**Keywords:** Geodynamics, Tectonics, Geophysics

## Abstract

Over geological time, the growth of the ocean floor involves magmatic and tectonic extension^1^ at mid-ocean ridges (MORs). Because seismogeodetic monitoring of these submarine plate boundaries remains challenging^2–7^, little is known about how these systems operate on yearly timescales. Here we report the first, to our knowledge, in situ observation of a rifting event at a MOR segment that combines hydroacoustic, direct-path ranging and bottom-pressure measurements, with repeated seafloor mapping. This event started on 26 April 2024 at the axis of the Southeast Indian Ridge (SEIR) near 37° S, two months after instruments had been deployed across the ridge axis and nearby Amsterdam transform fault (TF). The event began as a rapidly migrating swarm of extensional seismicity along the axial valley. It caused 4 m of subsidence of the valley floor and more than a metre of horizontal extension across the valley. We interpret this as the deflation of a sill-like reservoir feeding propagating dykes along the ridge axis. The dykes eventually led to the outpouring of about 160 million m^3^ of lava at the seafloor in about 16 days, while inducing both seismic and aseismic slip on valley-bounding normal faults and finally triggering seismic activity on the abutting TFs. Large-scale aseismic slip induced by magmatic processes could therefore be the primary mechanism by which MOR normal faults accrue their displacement, which would account for their well-documented seismic deficit^8,9^.

## Abstract (web summary)

By combining hydroacoustic, direct-path ranging and bottom-pressure measurements, in situ observations of a rifting event at a segment of the Southeast Indian Ridge are reported, providing insight into seafloor spreading events on yearly timescales.

## Main

Two-thirds of Earth’s surface was created along the 65,000-km-long MOR system, a network of narrow tectonic boundaries in which new lithospheric plates form and drift apart at a scale of centimetres per year. Under MORs, mantle rises and partially melts on decompressing. Buoyant magma ascends towards the ridge axis, where it crystallizes as new oceanic crust within spreading segments bounded by TFs or non-transform offsets. Magmatic emplacement, however, rarely accounts for all of the plate divergence and part of the extension is accommodated by slip on normal faults of all sizes^1^. Over hundreds of thousands of years, this interplay of magmatic and tectonic deformation shapes abyssal hills, which cover most of the ocean floor^10,11^. Little is known, however, on how these processes unfold on short timescales. Seafloor spreading is thought to proceed as a succession of ‘quantum’ events of extension, involving earthquakes and dyke intrusions^12^. These events can last several hours to several months and recur on decadal timescales, depending on the local spreading rate and magma supply. Many questions remain about how stresses accumulate between these events^13,14^, the degree to which activity on spreading segments and nearby TFs is synchronized^15^ and why MORs release so little seismic energy given their rapid time-averaged deformation rates^8,9^. These knowledge gaps originate from the challenge of simultaneously and continuously monitoring, for several years, the low-level seismic activity and the horizontal and vertical displacements of the seafloor along submarine plate boundaries^16,17^. We took on this challenge by installing an autonomous seismogeodetic observatory on an active MOR segment and its abutting TF and were lucky enough to capture a notable seafloor spreading event two months into our experiment.

## Setting of the OHA-GEODAMS experiment

**Fig. 1:**
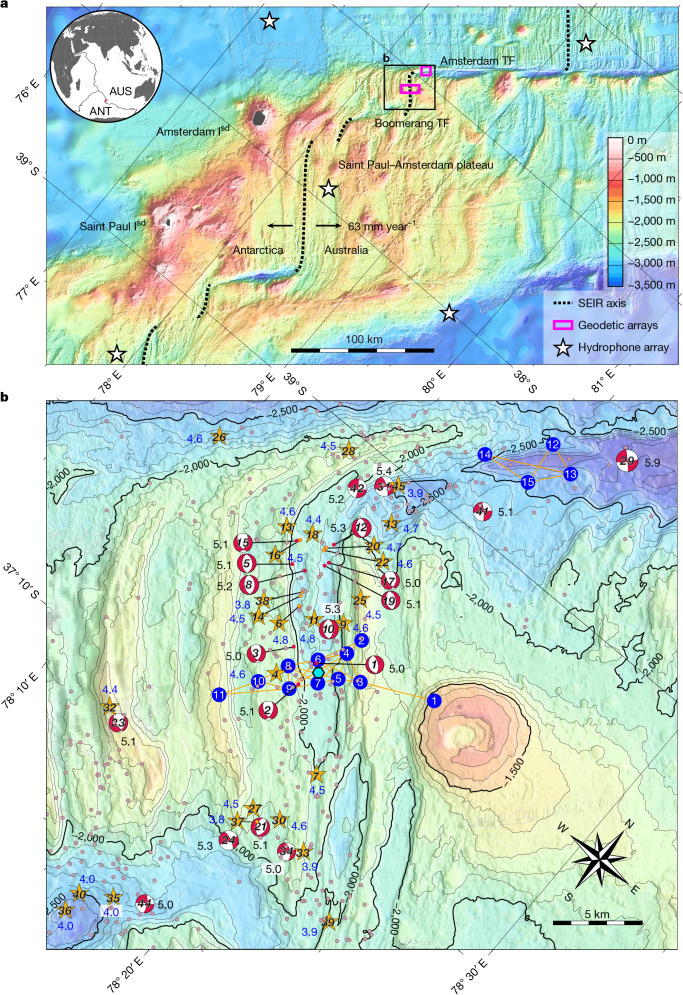
Tectonic setting and seismicity of the captured seafloor spreading and transform faulting event. **a**, This event occurred along the SEIR segment I1 (in the black rectangle), located on the Saint Paul–Amsterdam plateau in the Southern Indian Ocean (oblique Mercator projection). The seismogeodetic experiment included a wide array of autonomous hydrophones (stars), two arrays of acoustic ranging beacons and one BPR (in the purple rectangles) and repeated swath-bathymetry surveys of the axial valley and its intersection with the two abutting TFs. **b**, Earthquake crisis of 26 and 27 April 2024. GCMT focal mechanisms (red with Mw magnitudes in black) and ISC events (orange stars with mb magnitudes in blue) from land-based catalogues are numbered sequentially (italicized numbers; Extended Data Table 1) and were all relocated from the recordings of the hydrophone array, which also recorded many lower-magnitude hydroacoustic events (grey dots). Horizontal displacements were measured along direct-path ranging baselines (orange lines) between acoustic beacons (white numbers in blue circles) distributed in two arrays. Vertical displacement in the axial valley floor was measured with a self-calibrating BPR (cyan hexagon).

The OHA-GEODAMS (Observatory with Hydro-Acoustics and Geodesy near Amsterdam Island) observatory was deployed late February 2024 across the axis of the SEIR at 37° 07′ S (segment I1 (refs. ^18,19^)) and across the nearby Amsterdam TF (Fig. 1a). This region marks the northernmost extent of the Saint Paul–Amsterdam volcanic plateau, which the SEIR is now splitting apart^18–20^. The local spreading rate is 61 mm year^−1^ based on magnetic anomalies^21,22^ and 63 mm year^−1^ according to space geodesy^23^. The seafloor produced at segment I1 is typical of intermediate-spreading MORs, with an approximately 2,000-m-deep axial valley flanked by fault-bounded abyssal hills, hundreds of metres tall and spaced roughly every 2 km (Fig. 1b). This area experiences one to two Mw ≥ 5 earthquakes each year either on the Amsterdam TF or the Boomerang TF that bound segment I1 and has the advantage of being regularly visited by R/V Marion Dufresne on her yearly voyages to maintain the French scientific research stations in the Southern Indian Ocean islands.

Our observatory comprises an array of five autonomous hydrophones, encompassing the whole Saint Paul–Amsterdam plateau (Fig. 1a), to monitor the regional seismic activity. Passive acoustics (Methods) have proved to be very effective in locating low-magnitude seismic activity in MOR contexts, with a location accuracy of 1–2 km or better and completeness on the order of mb = 3.9 (refs. ^15,24–26^). They have been particularly successful at detecting seafloor spreading events involving dyke injection and eruption^27–30^. To monitor horizontal displacements at the ridge–transform intersection, we deployed 15 acoustic-ranging beacons or transponders (TP (refs. ^3,7,31^)) on the seafloor (Fig. 1b), 11 straddling the axial valley (TP1–TP11) and four across the Amsterdam TF (TP12–TP15). To make baselines as long as possible, the beacons were mounted on high tripods and placed on ridge crests and cliff edges. Across the axial valley, beacons were set along two parallel lines, 1.3 km apart with common end-stations, to extend the lateral coverage of the array and to ensure redundancy in case of instrument failure. Overall, the network spans 11 km, with baselines ranging in length from 1,100 to 4,300 m. Owing to the rugged and elevated terrain, not all beacons can communicate with all of their neighbours; still, a total of 32 baselines can be monitored by direct-path ranging. The TF array forms a diamond spanning the Amsterdam TF, with four 1,940–4,940-m-long baselines. Ranging measurements were repeated every 4 h in the ridge array and every 2 h in the TF array to measure the relative displacements between transponders (Methods). Finally, we deployed a drift-controlled bottom-pressure recorder (BPR) between transponders TP6 and TP7 (Fig. 1b) to monitor vertical displacements of the axial valley floor (Methods). Even though all of these approaches have been used previously to study ground deformation associated with seafloor spreading^2–7^, this is the first time that they have all been implemented simultaneously on a MOR segment and abutting TF exhibiting substantial tectonic activity.

## Seismicity and seafloor displacements

**Fig. 2:**
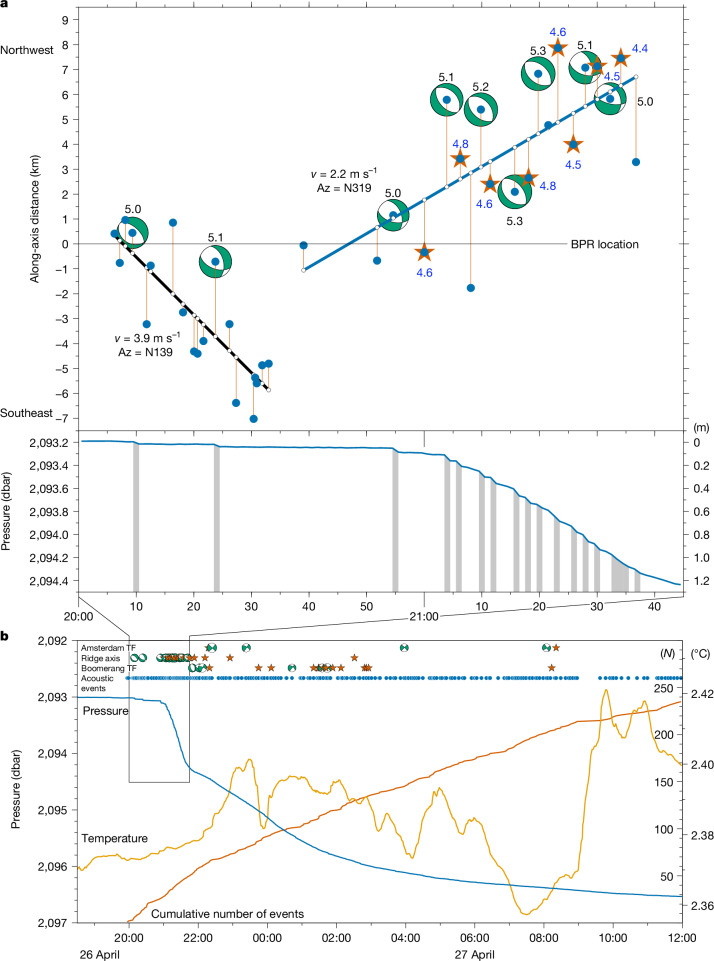
Initial sequence of seismic events and vertical displacements of the valley floor. **a**, Sequence of events in the axial valley interpreted as the result of dyke propagations, initially to the southeast (N139) and then to the northwest (N319), at an average speed of about 3 m s^−1^. Along-axis distances are relative to the BPR location. GCMT focal mechanisms (green), ISC (orange stars) and hydroacoustic events (blue dots) are shown with their Mw (black) and mb (blue) magnitudes, respectively. The lower part shows the BPR recordings of the coseismic subsidence of the valley floor as the dyke propagated; episodes of subsidence exceeding 1.7 cm min^−1^ are shaded. Map view of the events and dyke propagations is shown in Extended Data Fig. 9. **b**, Longer-term subsidence (blue curve, 1 dbar ≈ 1 m) and temperature changes (orange curve) recorded by the BPR in the axial valley floor. The framed area corresponds to the time window in panel **a**. The brown curve is the cumulative number (*N*) of seismic and hydroacoustic events reported at the top of the panel and distributed between the axial valley and the two adjacent TFs.

On 26 April 2024, at 19:56 UTC, a notable swarm of extensional seismicity began in the middle of segment I1 with five small events detected by the hydrophones, followed by a Global Centroid Moment Tensor (GCMT)^32^ Mw = 4.9 normal-faulting earthquake at 20:09, located under the axial valley (number 1 in Fig. 1b). This initial seismic activity, with a subsequent Mw = 5.1 and 16 lower-magnitude earthquakes, migrated more than 8 km towards the southeastern end of the axial valley until 20:35 (Fig. 2a). It was followed minutes later by a series of seven Mw ≥ 5 normal-faulting (GCMT), eight 4.8 > mb > 4.4 (International Seismological Centre (ISC))^33^ and four lower-magnitude earthquakes, which migrated more than 9 km from the segment centre to its northwestern end, in the direction opposite to that of the initial migration phase. Such migrating patterns are typical signatures of dyke propagation events along extensional plate boundaries^34–36^, although the migration rates involved in this case (2–3 m s^−1^, from the regressions shown in Fig. 2a) are an order of magnitude faster than those documented in analogous, albeit longer, sequences (Table 1 in ref. ^29^). Remarkably, the axial valley floor is 200 m shallower and narrower at the southeastern end of the segment compared with its northwestern end (Fig. 1b). A southeastward dyke intrusion from the segment centre may thus have been hindered by topographic stresses^37^, giving way to a more mechanically favourable northwestward intrusion.

**Extended Data Fig. 1:**
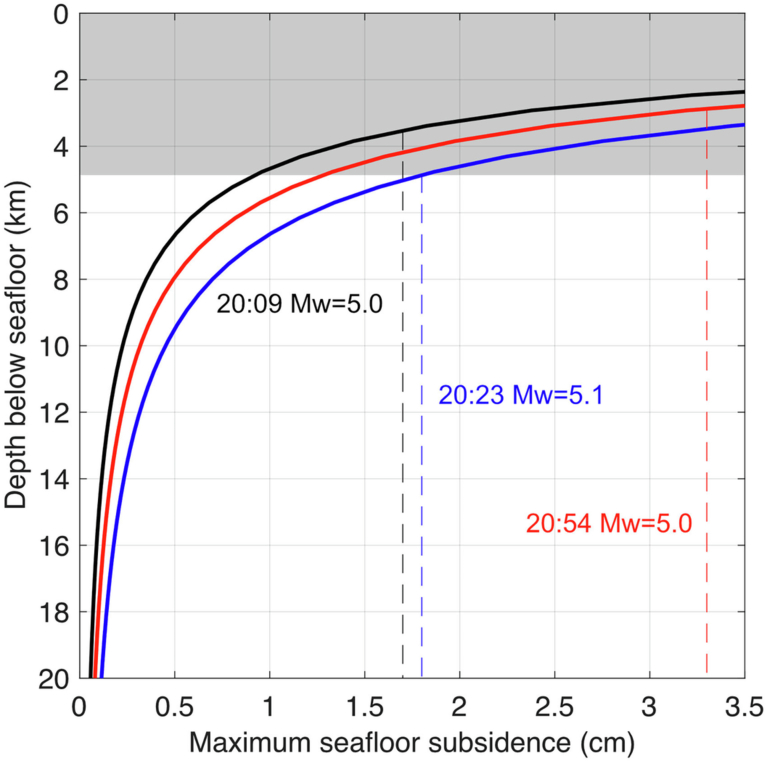
Constraint on the depth of the first three Mw ≈ 5 events from coseismic seafloor subsidence. Depths below the seafloor cannot exceed about 5 km (greyed area), as inferred from the rupture size (derived from Mw) and the coseismic subsidence (Fig. 2a) recorded by the BPR in the axial valley (cyan hexagon in Fig. 1).

In the meantime, the BPR in the axial valley floor recorded a series of discrete subsidence events, that is, sudden pressure increases amounting to between 1.7 and 3.3 cm of vertical motion coincident with each of the first large normal-faulting events (Fig. 2a). Using the rupture size inferred from the GCMT magnitude^38^ and fault dips given by the focal mechanisms, we find that these earthquakes, modelled as circular ruptures in an elastic half-space (Methods), could not have occurred any deeper than about 5 km below the seafloor to cause the observed coseismic subsidence (Extended Data Fig. 1). These markedly shallow depths are in strong contrast to those estimated by the GCMT catalogue (12–16 km below the seafloor), probably because of the poor teleseismic coverage of the area.

**Extended Data Fig. 2:**
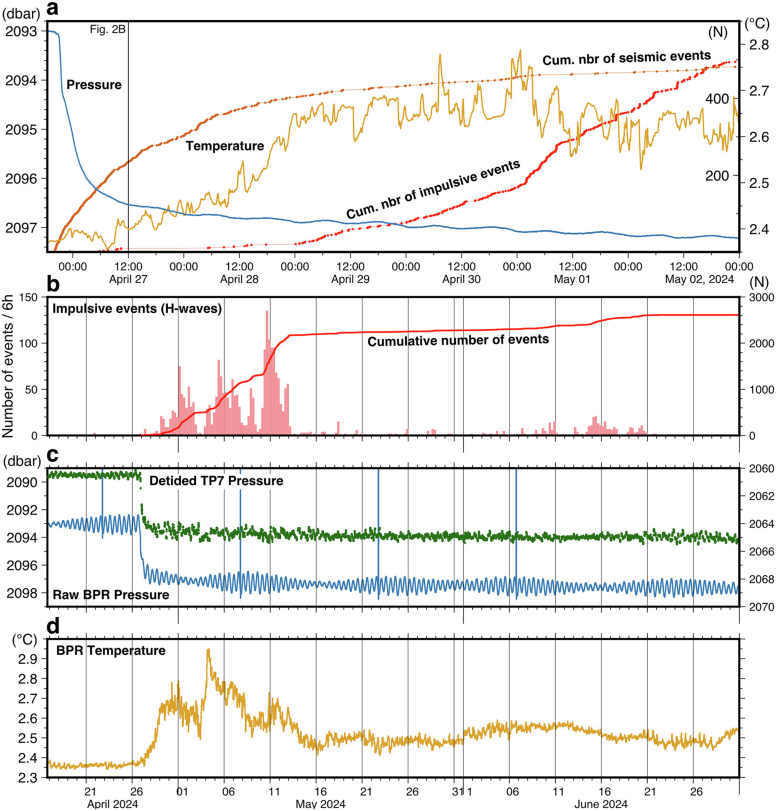
Longer-term records of the drift-controlled bottom pressure, temperature and impulsive events. **a**, Pressure (blue) and temperature (orange) recorded by the BPR versus cumulative number (*N*) of seismic events (brown) and impulsive events (red) recorded by four hydrophones. Note the residual tide signal in the 4.2-m-high recorded subsidence (that is, 4.2 dbar pressure increase) and the gradual increase of the sea-bottom water temperature as the number of impulsive H-wave events reflecting hot lava–seawater interaction increases. **b**, Histogram (6-h bins) and cumulative number of the impulsive events (H-waves) marking the onset and demise of the lava extrusion. They occurred in three main pulses between 26 April and 12 May 2024, with a minor burst on 15 and 16 June 2024. These events have been automatically picked on three to four hydrophones and associated for trilateration. **c**, Longer-term raw BPR pressure data (blue, left axis) showing the cyclical tide and the self-calibrating measurements (vertical bars; Methods) and pressure measured at the TP7 beacon corrected for the tide (green, right axis). TP7 and the BPR, 690 m apart (Fig. 3), both subsided by more than 4 m. **d**, Long-lasting temperature (orange) anomalies recorded near the sea bottom by the BPR.

Subsidence of the axial valley floor accelerated after a Mw = 5.1 event at 21:03 (number 5 in Fig. 1b), in a stepwise manner, averaging about 5 cm min^−1^ while the putative dyke propagated to the northwest. When the propagation stopped at about 21:40, cumulative seafloor subsidence had reached 1.2 m (Fig. 2a). Subsidence then slowed abruptly and continued at a lesser rate of about 0.6 cm min^−1^ until 01:30 on 27 April (Fig. 2b). It slowed further to 1.2 cm day^−1^ until 00:00 on 3 May and beyond (Extended Data Fig. 2). The total subsidence amounted to 4.2 m over a period of 6 days, of which 83% occurred in the first 16 h. This is almost twice the total subsidence of 2.45 m measured over 12 days during the 2015 eruption of Axial Seamount, of which 91% occurred during the first 24 h (ref. ^17^). Like at Axial Seamount, we interpret this vertical displacement of the valley floor as resulting from the drainage of an underlying magma reservoir (for example, axial melt lens^39^). The rapid subsidence between 21:03 and 21:40 may specifically represent the drainage from the magma reservoir into a northwest-propagating dyke. Rising near-bottom water temperature recorded by the BPR suggests that lava may have reached the seafloor as early as 22:00 (Fig. 2 and Extended Data Fig. 2). Past that time, slower seafloor subsidence may reflect magma drainage through open fractures connecting the reservoir to the seafloor.

**Fig. 3:**
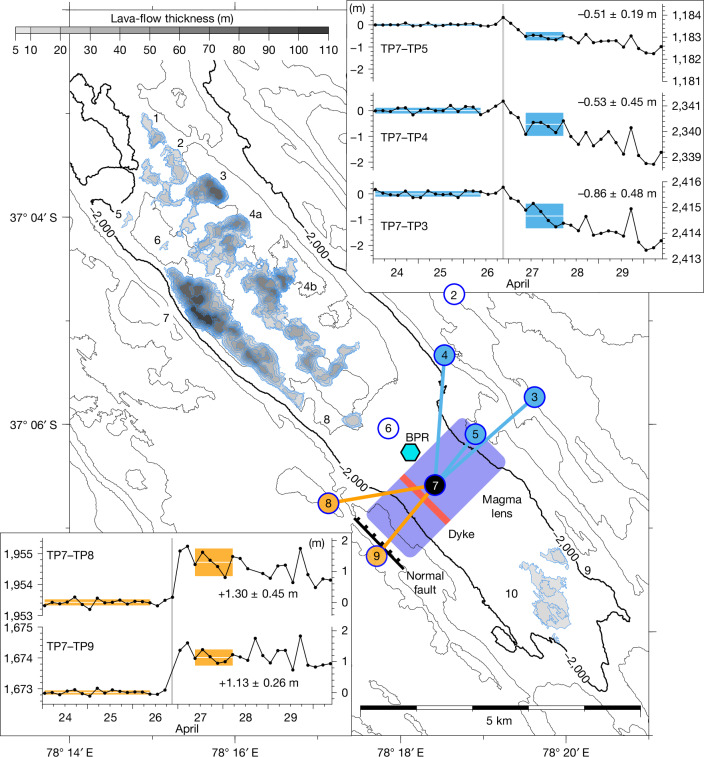
Initial baseline changes in the axial valley, and extent and thickness of the new lava flows. Displacements in metres are measured from transponder TP7 along the coloured baselines to the other transponders (numbered circles), which are either lengthening (orange) or shortening (blue). Greyed patches with 10-m contours in light blue show the extent of the new lava flows, which exceed a thickness of 90 m in places (see greyscale in the upper left; volumes of patches 1 to 10 are given in Extended Data Fig. 6). Insets show the initial changes in the baselines with up to 1.30 m extension to the southwest (TP8, TP9) and 0.86 m contraction to the northeast (TP3, TP4, TP5). Coloured rectangles show averaging windows and 1*σ* uncertainty. The vertical grey line corresponds to the time of the rapid subsidence of the BPR (between 21:00 and 21:40 on 26 April; Fig. 2b). The baseline changes are interpreted as the combined result of a propagating dyke and a deflating magma lens at depth below TP7 (purple rectangle) and normal slip along a fault bordering the axial valley and dipping 60° inwards towards the dyke (Fig. 4). Longer-term changes in the baselines are shown in Extended Data Fig. 3.

**Extended Data Fig. 3:**
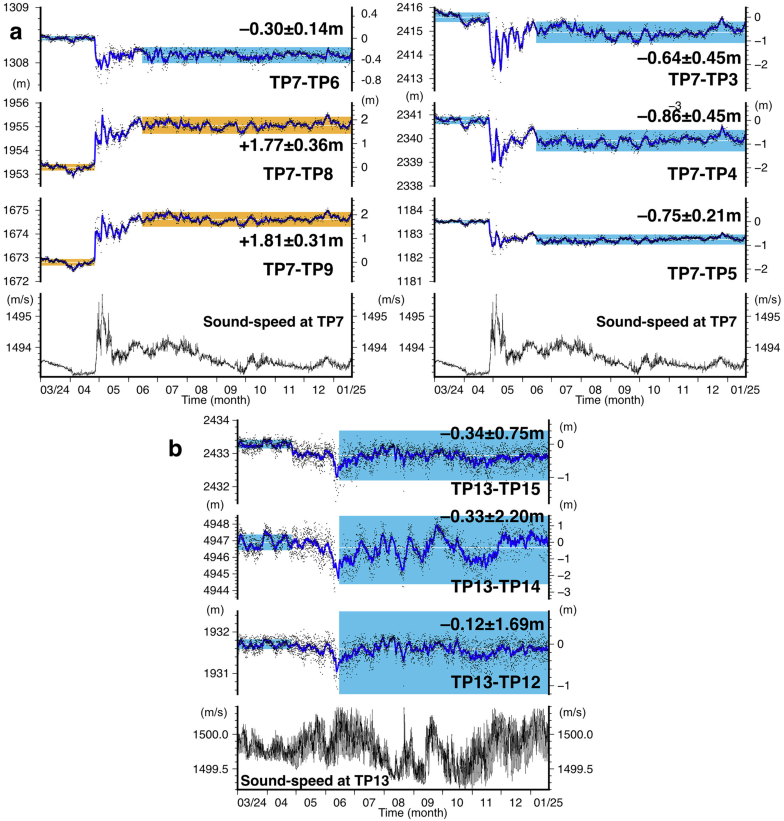
Longer-term evolution of the direct acoustic-ranging baselines across the axial valley and the Amsterdam TF. **a**, Baselines (blue curves) from transponder TP7 to transponders TP3–TP4–TP5–TP6–TP8–TP9 in the axial valley (ridge array) are in either extension (orange) or contraction (blue). Vertical axes: actual baseline length (left) and relative changes (right). Bottom plots show the evolution of the sound speed at TP7, on which the ranging measurements are based, and which varies, owing to increases in the sea-bottom water temperature caused by the lava extrusions (Extended Data Fig. 2d). **b**, Baselines from transponder TP13 to transponders TP12–TP14–TP15 on either side of the Amsterdam TF (TF array). There is no sign of clear strike-slip displacement along the TF; data are obscured by unexplained cyclical changes in the sound speed at TP13 (bottom plot). Uncertainties after the event are large owing to the important tilt of all tripods caused by the series of large-magnitude earthquakes.

Nearly concurrently, the direct-path acoustic ranging array straddling the axial valley recorded substantial horizontal displacements (Fig. 3). During the February 2025 maintenance cruise, given the challenge to reposition the beacons on the seafloor, only the central station TP7 was recovered to access its full dataset. Baselines remained fairly constant until the ranging session at 21:12 on 26 April, which nearly coincided with the Mw = 5.2 normal-faulting event at 21:09 (number 8 in Fig. 1b), at the beginning of the rapid subsidence phase of the BPR. Afterwards, baselines markedly changed but fluctuated about their new lengths, probably because of rapid changes in sound speed induced by fluctuating ambient temperatures (Fig. 2b). The temperature field was then certainly not uniform near the axial valley floor (that is, around TP7) and affected all acoustic travel times to the elevated beacons on the valley walls. Nevertheless, by averaging the ranging measurements before the ambient temperature increased by more than about 0.1 °C, baselines from station TP7 to the two stations located on top of the southwest valley wall (TP8 and TP9) clearly lengthened by up to 1.30 m, whereas, on the opposite side of the valley, baselines shortened by 0.53 m (TP4 and TP5) and 0.86 m (TP3; averaging windows shown in Fig. 3). These displacements are one order of magnitude larger than those measured across the Juan de Fuca Ridge at 46° N during the 25 January 1998 dyke intrusion at Axial Seamount^4,31^. However, our baselines do not show any obvious extension related to some inflation before the 26 April events, as was observed at Axial Seamount. All tripods tilted by several degrees during the swarm of nearby Mw ≥ 5 quakes. Because we cannot tell in which direction they tilted, the uncertainties in the baseline changes include the worst-case scenario (that is, acoustic heads fully tilting apart or closer). Extension between TP8–TP9 and TP7 continued until June 2024 and reached up to +1.80 m, whereas TP3–TP4–TP5 ended up closer to TP7 by −0.64 to −0.86 m (Extended Data Fig. 3). Notably, baseline TP6–TP7 also diminished. This could be because of the subsidence of the two transponders, as recorded by the built-in pressure gauge in TP7 (Extended Data Fig. 2c), possibly suggesting that the deflation of the magma reservoir was centred below the BPR and TP7 (Fig. 3), which both subsided by about 4 m.

**Extended Data Fig. 4:**
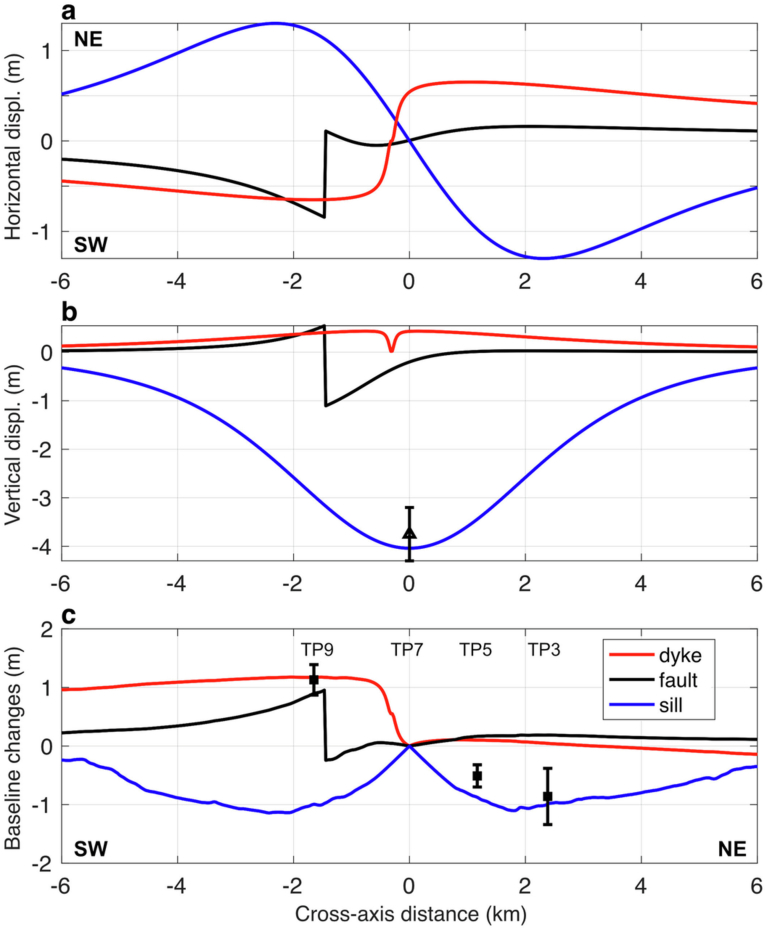
Components of the horizontal and vertical displacements, and baseline changes in the model of Fig. 4, relative to TP7 (*x* = 0). **a**, Horizontal displacements (positive towards northeast, negative towards southwest). Compaction of a magma lens below TP7 (that is, deflation of a reservoir owing to magma withdrawal) generates contraction on either side of TP7 (roughly within ±2 km) and extension further outward. The dyke opening generates extension southwest of TP7, whereas the fault slip generates about 1 m of extension at the apex of the fault. **b**, Vertical displacements. The drainage of a magma lens (sill) accounts for most of the vertical subsidence measured at TP7 (triangle with error bar). It overwhelms the moderate uplift owing to the dyke emplacement. The fault slip generates 1.0 m of subsidence at its foot and a 0.5 m uplift of its shoulder. **c**, Contribution of each component to the observed baselines changes relative to TP7 (symbols with error bars). Combining all contributions—sill deflation, dyke opening and fault slip—yields the fit shown in Fig. 4 (with a total RMS misfit of 17 cm).

The initial displacements (Fig. 3) along a cross-axis line joining TP9 to TP3, through TP7 and TP5, provide critical constraints to evaluate the interplay of different tectonomagmatic deformation sources during the early stages of the spreading event, that is, fault slip, deflation of a magma reservoir and intrusion of a dyke. Station TP7 subsided by 3.75 m ± 0.55 m, whereas TP9 moved away from TP7 and TP5 and TP3 moved closer to TP7. Such massive subsidence is readily explained by a deflating magmatic sill^40^ that would also induce horizontal contraction bringing all transponders closer to TP7 (Extended Data Fig. 4). Lengthening of the baselines thus requires sources of extension between TP9 and TP7, such as the opening of a dyke and/or slip on a normal fault.

**Fig. 4:**
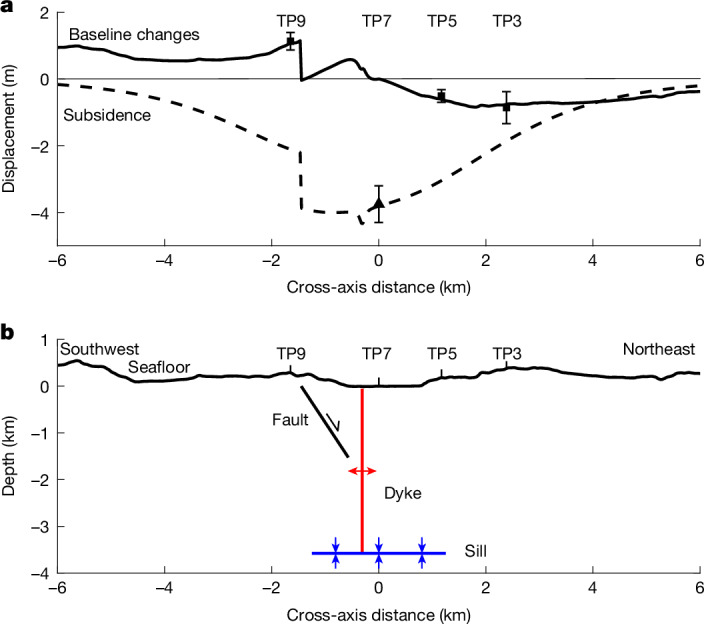
Example of a 2D deformation model fitting the observed vertical displacements and baseline changes relative to TP7 along a profile perpendicular to the axial valley. **a**, Overall baseline changes (Δ*L*; Methods; continuous line) and vertical subsidence (dashed line). The overall horizontal displacement is 2.4 m (60% dyke, 40% fault). **b**, Geometry of the model components below a bathymetric cross-section: a 3,577-m-deep sill deflating by 9.8 m, a 1.4-m-wide dyke rising 3,520 m from the sill up to 57 m below seafloor, between TP9 and TP7 (310 m southwest of TP7) and 1.9 m of slip down to 1,526 m depth on a northeast-dipping normal fault (total RMS misfit = 17 cm). A schematic map view of the sill, dyke and fault is shown in Fig. 3. The individual contribution of each component—sill, dyke and fault—to the displacements is shown in Extended Data Fig. 4. This is one of the 2,203 solutions best fitting the data (RMS misfit < 20 cm) out of 10 million randomly sampled models. The only fixed parameters were the fault dip set at 60°, based on focal mechanisms, the fault location along a southwest valley wall between TP7 and TP9 and the sill width across axis set at 2,500 m and centred on TP7 (Methods). Coloured arrows show the expected type of motion on the fault, dyke and sill.

**Extended Data Fig. 5:**
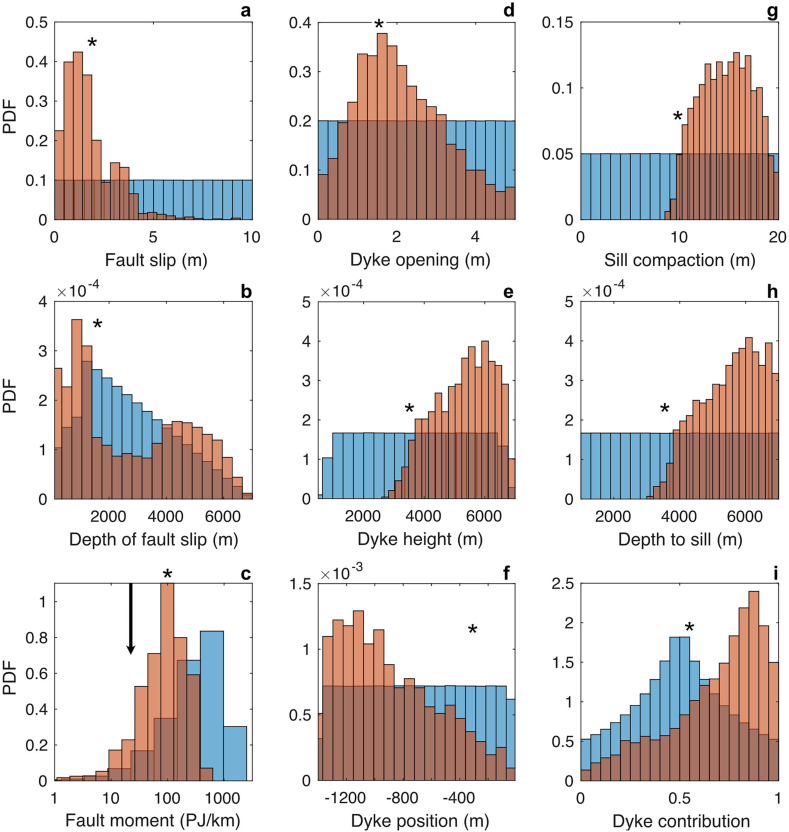
Probability density function of the elastic model parameters for the 2,203 best-fitting solutions (out of 10 million random models). **a**–**i**, Asterisks (*) correspond to the model shown in Fig. 4. Blue histograms are the initial probability density functions (PDFs); brown histograms show the resulting range of best solutions (RMS misfit < 20 cm) for each of the inverted parameters. Horizontal axes give the full range of the seven variables tested in the models: fault slip (panels **a** and **b**), dyke (panels **d**–**f**), and sill (panels **g** and **h**). Fault moment in panel **c** is derived from panels **a** and **b**. Dyke contribution to the total extension in panel **i** is derived from panels **a**, **b** and **d**, both for a fault dipping 60°. The arrow in panel **c** indicates the moment released by the series of Mw ≈ 5 earthquakes in the axial valley (22.6 PJ km^−1^), which is a small fraction of the total moment predicted by the models best fitting the measured displacements. The remainder reflects the moment taken up by aseismic slip during this quantum spreading event.

Using 2D elastic dislocation models (Methods), we randomly sampled 10 million combinations of sill, dyke and fault geometries to assess how well they could account for the observed displacements (Extended Data Fig. 5). Of these, 2,203 yielded a satisfactory root mean square (RMS) misfit (<20 cm). They have in common: (1) a sill at least 3,500 m deep collapsing by 12–18 m; (2) a dyke rooted at the sill, extending to sub-seafloor depths of 83–377 m and opening by 1.0–3.3 m; (3) 0.6–3.1 m of slip on a normal fault down to a few kilometres; and (4) a total horizontal extension of 2.1–4.0 m, distributed between the dyke and the fault. Most models favour the dyke taking up more extension than the fault. As an illustration, Fig. 4 presents a plausible model with a sill, 2,500 m wide and 3,600 m deep, deflated by 9.8 m; a dyke rising to 57 m depth beneath the geodetic network and opening by 1.4 m; a northeast-dipping axial-valley bounding fault slipping by 1.9 m down to 1,500 m depth. Such a large amount of slip, one order of magnitude larger than expected from any Mw ≈ 5 earthquakes of the swarm (Methods), implies that the fault motion largely occurred aseismically. In this particular scenario, the total horizontal extension amounts to 2.4 m (60% dyke, 40% fault), which is equivalent to 38 years of spreading at 63 mm year^−1^.

## New lava flows on the seafloor

**Extended Data Fig. 6:**
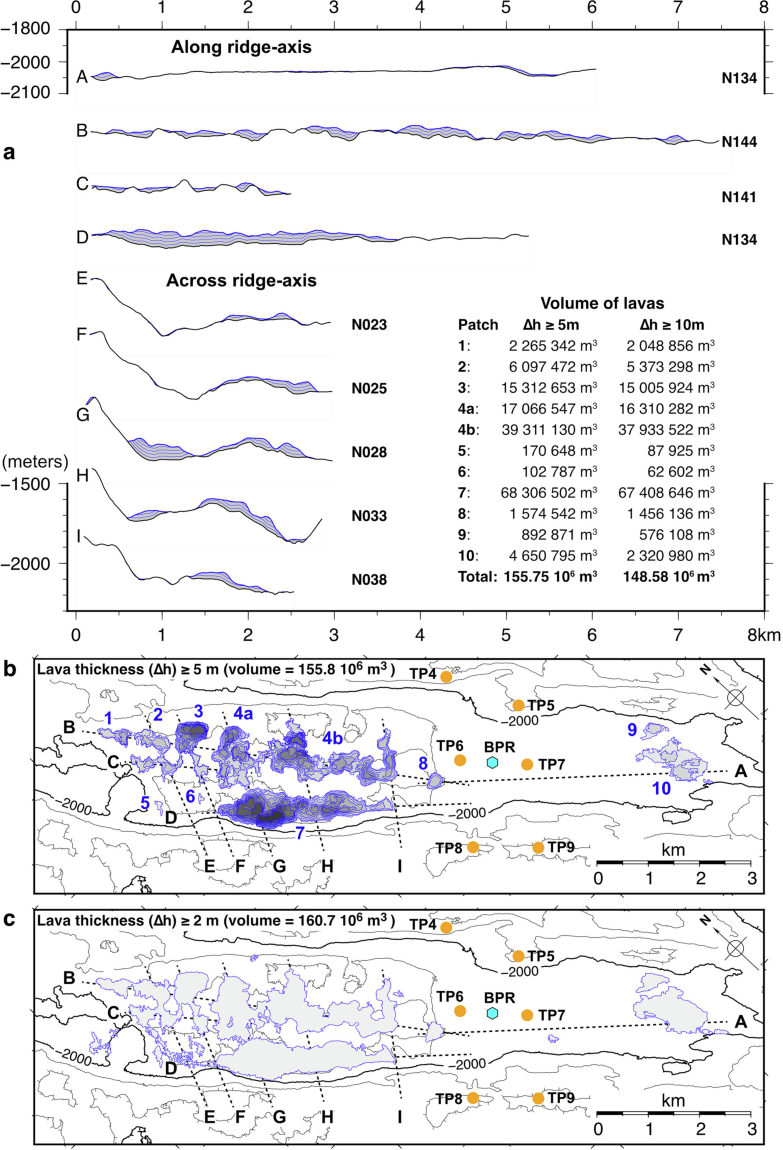
Cross-sections and volumes of the new lava flows. **a**, Across-axis sections (E to I) clearly show that lavas originated from two distinct sources, one centred on the former axial ridge, probably fed by the northwest-propagating dyke, and the other located at the foot of the valley wall, probably rooted to the dyke through the axial-valley bounding fault. Sections H and I show that, in places, lavas flowed down the slopes and filled depressions. All cross-sections are at the same vertical scale, with isopach lines 20 m apart. **b**, Isopach map of the lava flows thicker than 5 m (same colour scale as in Fig. 3). **c**, Extension of the lava flows thicker than 2 m. Whether we consider elevation differences (Δ*h*) greater than 2, 5 or 10 m to be notable, between the 2024 and 2025 bathymetric grids, the total volume of the new lava flows ranges between 149 and 160 million m^3^. Isobaths on the maps every 100 m.

During the deployment of the observatory (2024) and first maintenance cruise (2025), we repeated a ship-based swath-bathymetry mapping of the axial valley, its flanks and its connection with the Amsterdam TF. To compensate for the lack of high-resolution mapping near the seabed, we collected several overlapping swaths at reduced speed during each leg. Subtracting the digital terrain models (DTMs)^41,42^ from the 2024 and 2025 surveys revealed marked changes in valley-floor topography (Fig. 3), despite the low resolution (20 m) of the models with depth-sounding uncertainties up to 5 m. Relative to 2024, the 2025 survey mapped large areas with positive depth changes, locally exceeding 90 m. We interpreted these areas as the outlines of substantial new lava flows along two distinct parallel lines. The thickest one, forming a 4-km-long patch (number 7 in Fig. 3), outpoured at the foot of the southwestern valley wall, which experienced several large normal-faulting events. The second line of massive lava flows forms a series of distinct or connected patches (numbered from 1 to 4) aligned with the axial volcanic high that was mapped along the ridge axis in 2024. A 40-m-tall circular patch (number 8) formed only 600 m away from beacon TP6. The northwest-propagating dyke probably fed the central line of lava flows but also supplied magma that possibly extruded through the western fault plane. Two other patches (numbers 9 and 10, with thicknesses up to 20 m) are located at the southeastern end of the segment and were probably fed by the early southeast-propagating dyke. The total volume of new lavas ranges from 148 to 160 million m^3^ (Extended Data Fig. 6), in the range of lava emplacements reported in comparable MOR contexts (for example, the Galápagos, Gorda, Juan de Fuca ridges; Fig. 12 in ref. ^43^).

**Extended Data Fig. 7:**
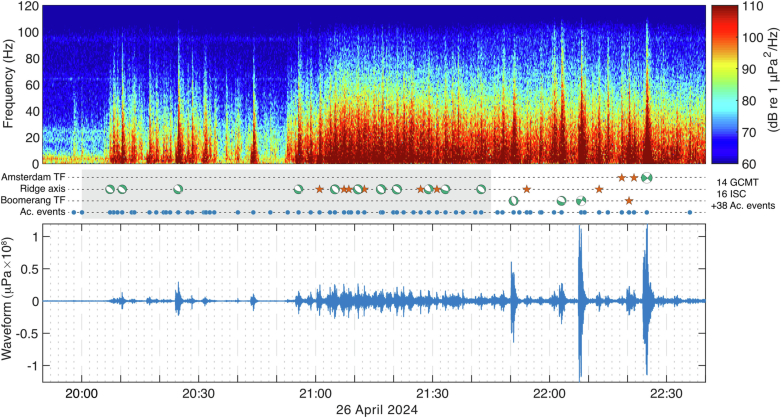
Hydroacoustic spectrogram and waveform at the central hydrophone (central star in Fig. 1a) showing the onset of the 26 April 2024 seafloor spreading event and the sharp increase of T-wave and H-wave events after 20:50. The middle panel shows the arrival times of the events shown in Fig. 1b and their distribution between the ridge axis and the two abutting transforms. The shaded area corresponds to the dyke propagation shown in Fig. 2a.

The timing of lava effusion can be constrained through indirect evidence: (1) the change of slope in the rapid subsidence phase of the BPR, which coincides with a gradual increase of the sea-bottom temperature starting on 26 April at 22:00 (Fig. 2b and Extended Data Fig. 2) and (2) the occurrence of numerous short (<10 s) and energetic hydroacoustic events, called H-waves and generated in the water column by lava–seawater interactions^16,44–47^ (Extended Data Fig. 2). These events begin around 06:00 on 27 April. There may have been earlier ones, masked by the intense seismic activity recorded by the hydrophones (Extended Data Fig. 7). H-wave events greatly increased after 27 April. More than 2,150 were recorded by at least three hydrophones by 12 May, after which they stopped, suggesting that the eruption lasted only 16 days, thus with an average rate of 9–10 million m^3^ of lava extrusion per day (effusive rate of 116 m^3^ s^−1^) and a final short outburst on 15 and 16 June 2024. The three main pulses of H-wave events match increases in the sea-bottom temperature at the BPR (Extended Data Fig. 2). Their sources cluster around the northern lava flows, which are also the most voluminous. At Axial Seamount and the East Pacific Rise, the occurrence of these impulsive events were similarly interpreted as marking the onset and duration of lava extrusion^16,44,45^.

## Transform activation and strain budget

**Fig. 5:**
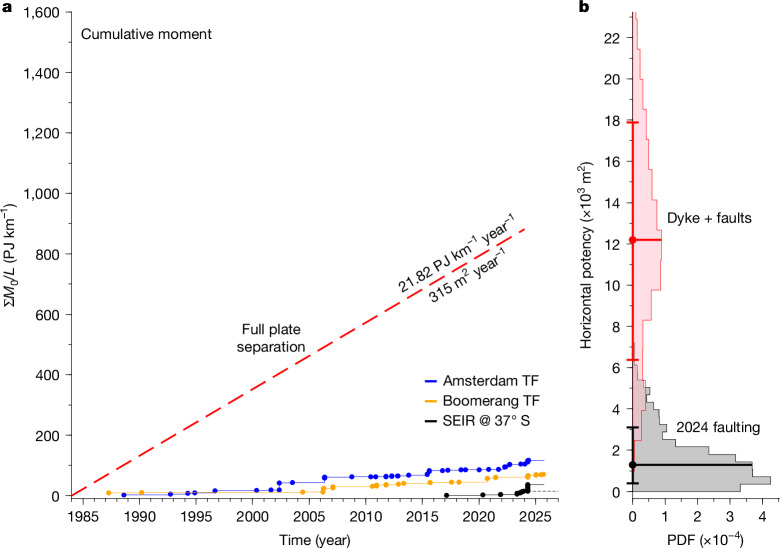
Cumulative moment budget along the Amsterdam and Boomerang TFs and spreading segment in between (37° 05′ S, segment I1) based on the GCMT catalogue^32^ and horizontal potency^48^ of the 2024 spreading event. **a**, Seismic moments (*M*_0_), expressed in 10^15^ J or PJ, have been normalized by the length (*L*) of the TFs and ridge segment: 115, 54 and 25 km, respectively. The dashed line indicates the expected horizontal potency rate (and its equivalent moment rate for a fault dipping at 60°) for a ridge segment continuously spreading at 6.3 cm year^−1^. **b**, Probability density function (PDF) of the equivalent horizontal potency^48^ released by fault slip—seismic and aseismic (grey histogram; modelled in Extended Data Fig. 5c); relative to the median normal-faulting moment (black symbol with range corresponding to the 16th and 84th percentiles), it shows that the 2024 event was about 24% seismic and about 76% aseismic. The same quantity can be estimated for the dyke as the product of its opening by its height (Extended Data Fig. 5d,e). These distributions can be combined to estimate the total horizontal potency of the 2024 spreading event (tectonic + magmatic; red histogram); the median estimate of 12.3 × 10^3^ m^2^ (red symbol ≈ 850 PJ km^−1^) would correspond to the geometric moment accumulated over about 39 years of divergence of the SEIR at 6.3 cm year^−1^.

**Extended Data Fig. 8:**
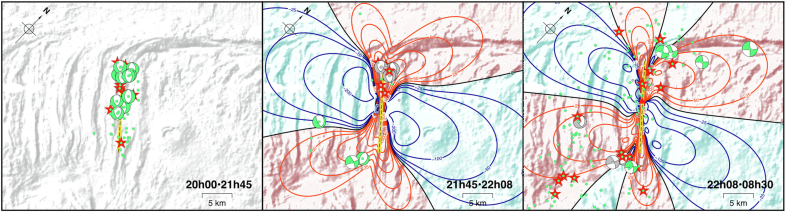
Main phases of the 26 and 27 April 2024 seafloor spreading event and induced changes in Coulomb stresses. Left, initial dyke propagation (gold line) triggered by and/or inducing normal faulting along the walls bordering the axial valley. Background is the shaded bathymetry (oblique Mercator). Dyke propagation later triggered the reactivation of the southwestern diffuse Boomerang TF (centre), as predicted by the induced changes in Coulomb stresses, and ultimately reactivated the northeastern Amsterdam TF (right). Positive and negative Coulomb stresses, shown in red and blue colours, respectively, with contours in kPa, are calculated for strike-slip receiver faults oriented N024 in the central panel (Boomerang TF) and N049 in the right panel (Amsterdam TF) and for a propagating dyke 1 m wide. Coloured seismic (GCMT, ISC, stars) and hydroacoustic (dots) events occurred in the time windows shown; greyed symbols are events preceding the starting time of each panel.

Shortly after the dyke stopped propagating northward (21:40 on 26 April), seismic activity started in the area of the Boomerang TF with unexpected off-axis normal-faulting events (numbers 21 and 23 in Fig. 1b), with fault planes oblique relative to the strike of the axial valley, as well as strike-slip events (number 24 in Fig. 1b). Twenty minutes later (at 22:23), the Amsterdam TF produced several strike-slip earthquakes, including a notable Mw = 5.9 event (number 29 in Fig. 1b). This intense activity resulted in substantial tilts among the transponder array straddling the TF. Together with large cyclical changes in the sound speed, this tilting hindered our ability to detect any resolvable change in the length of baselines (Extended Data Fig. 3b). Overall, the seismicity timeline (Fig. 2b) strongly suggests that the rupture of the Boomerang TF, followed by that of the Amsterdam TF, was triggered by the dyke propagation along the adjacent ridge axis. A similar causal relationship was inferred following the 2006 eruption at the East Pacific Rise at 9° 50′ N (ref. ^15^). Estimation of Coulomb stress changes (Methods) imparted by a dyke on strike-slip receiver faults representative of the Boomerang and Amsterdam TFs supports this hypothesis (Extended Data Fig. 8). Altogether, the transform seismicity triggered by the 2024 seafloor spreading event amounts to about 12 PJ km^−1^ of seismic moment release on the Amsterdam TF and about 3 PJ km^−1^ along the Boomerang TF (1 PJ = 10^15^ J). These values are not unusual compared with earlier bursts of moment release, which have been occurring regularly on these two TFs for several decades, as recorded by the GCMT catalogue^32^ (Fig. 5).

**Extended Data Fig. 9:**
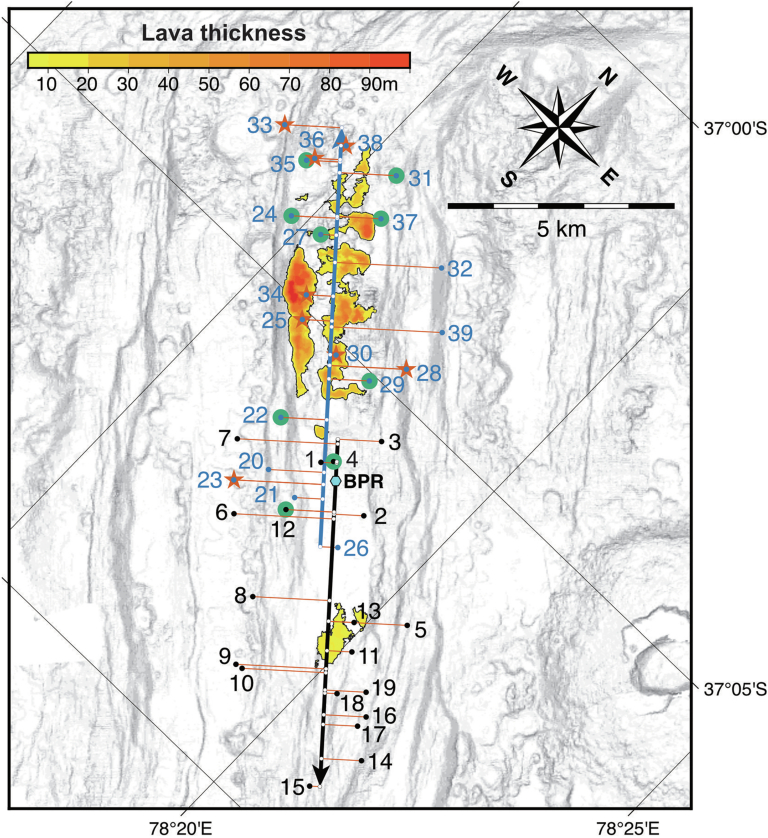
Map view of the two early dyke propagations shown in Fig. 2a (oblique Mercator view). Numbers give the event relative chronology, in black for the southeastward propagating dyke and in blue for the northwestward propagating dyke (stars, ISC; green circles, GCMT; dots, AUH catalogues). Coloured patches are the new lava fields (>5 m thickness); background image outlines the slopes of the seafloor.

By contrast, the approximately 25-km-long I1 spreading segment released no detectable normal-faulting moment before 2017. Between 2017 and 2023, it released about 14 PJ km^−1^ and the 2024 migrating swarm events (Extended Data Fig. 9) accounts for an extra approximately 22.6 PJ km^−1^. Notably, about 98% of the elastic models that fit our geodetic observations involve a normal-faulting moment larger than 22.6 PJ km^−1^ (brown histogram in Extended Data Fig. 5c). The median normal-faulting moment of these models is 92.3 PJ km^−1^ (range 29.1–212.9 PJ km^−1^, corresponding to the 16th and 84th percentiles, capturing 68% of the probability mass). By definition, this ‘geodetic’ moment includes both a seismic and an aseismic component, which, given our median estimate, implies that fault slip during the 2024 event was about 24% seismic and about 76% aseismic. This demonstrates the ability of MOR normal faults to experience aseismic slip events, which partly explains the well-documented deficit of extensional earthquakes in these settings^8,9^.

Another main contributor to this deficit is the fact that MOR normal faults only accommodate a fraction (1 − *M*)^11^ of the total plate divergence over geological time. Our results now show how this phenomenon manifests itself on a decadal timescale. Extending a 5-km-thick lithosphere at 63 mm year^−1^ amounts to a horizontal geometric moment rate of 315 m^3^ year^−1^ per metre (that is, m^2^ year^−1^) along axis. The geometric moment, also known as potency^48^, is a kinematic quantity that, unlike the scalar moment, allows for the direct inclusion of the contribution from dykes and faults. Our median estimate for the horizontal potency released by fault slip (seismic + aseismic) during the 2024 event, assuming a dip of 60°, is 1.3 × 10^3^ m^2^. The same quantity can be estimated for the dyke, as the product of its opening by its height (Extended Data Fig. 5d,e), yielding a median estimate of 10 × 10^3^ m^2^. Combining these distributions yields the total horizontal potency of the 2024 spreading event (tectonic + magmatic), with a median estimate of 12.3 × 10^3^ m^2^ (range 6.4–17.9 × 10^3^ m^2^; red histogram in Fig. 5). This amounts to the geometric moment accumulated during 39 years (range 20–57 years) of divergence at the SEIR.

We conclude that spreading segments undergo decades of quiescent stretching as abutting transforms accrue displacements by frequent slip events. The stresses that build up in the MOR segment are released during quantum events of seafloor spreading that involve both dyking and fault slip, including notable aseismic slip on axis-bounding normal faults, akin to a caldera collapse event, such as the Kīlauea’s 2018 collapse^49^. This may be the primary mechanism by which MOR normal faults accumulate displacement, potentially explaining their seismic moment deficit in global catalogues^8^.

## Methods

### Seismicity

Large-magnitude events were extracted from the GCMT catalogue^32^ and from the ISC bulletin^33,50^. All events were relocated from our hydroacoustic records (Extended Data Table 1).

### Hydroacoustics

The hydroacoustic array consisted of five autonomous hydrophones (Fig. 1a) moored in the axis of the SOFAR (sound fixing and ranging) channel and anchored to the seafloor. They continuously record sounds in the 1–125-Hz frequency band (250 Hz sampling rate), which include acoustic waves generated by seismic events near their epicentres^24,27^ (tertiary waves or T-waves) or by hot lava–seawater interactions (short impulsive events called H-waves)^16,44–47^, among other sounds (icequakes, large baleen whale calls or big vessels^25^). Source events can be precisely located (within 1–2 km)^15,24,25^ by trilaterating the T-wave or H-wave arrival times at each hydrophone, all synchronized with the GPS time. Following the approach of Fox et al.^24^, with at least four arrival times, we can derive the uncertainties in latitude, longitude and origin time from the covariance matrix of the nonlinear least-squares inversion. In that paper, they do not exceed 2 km (major axis) versus 1 km (minor axis) at 1*σ* (68% confidence level) inside the array; also, for each event, we systematically performed 10,000 random Monte Carlo draws within 1 s and 1 m s^−1^ intervals from the arrival times and sound speeds, respectively, yielding the same figures. Automatic event detection, arrival time association and trilateration are based on newly developed methods^51,52^. Unfortunately, the hydrophone located northwest of Amsterdam Island (top-left in Fig. 1a) failed in early April 2024. All reported events were consequently located from the acoustic records of the four remaining hydrophones. Hydroacoustic recordings were crucial in accurately repositioning the GCMT focal mechanisms and ISC events, provided in land-based catalogues with location uncertainties of 20 km or more owing to the remoteness of the area. Between the start of seismic activity on 26 April until 2 May 2024, nearly 500 T-wave events were identified and located (versus 22 GCMT and 52 ISC events) and more than 2,000 H-wave events (Extended Data Fig. 2).

### Direct-path ranging

Horizontal displacements of the seafloor were measured by acoustic direct-path ranging between Exail-manufactured Canopus beacons deployed on the seafloor. All acoustic transponders were mounted on a 3.50-m-high tripod and equipped with pressure, temperature and, for some, conductivity sensors to infer the sound speed at ranging times. During the maintenance cruise in 2025, limited datasets were downloaded with an acoustic modem to ensure that all beacons were running properly (inclination, battery level, sensors, some ranging measurements). The direct-path ranging schedule was designed to ensure that the batteries of the beacons would last 4 years. Accordingly, each station in the ridge array ranged to the neighbouring stations in acoustic line of sight every 4 h. In the TF array, ranging sessions were set 2 h apart. Hence, during a ranging session, all baselines are measured twice, that is, both ways, with a precision of 1.5 microseconds for the two-way travel times (<3 mm at 1,500 m s^−1^). Travel times were then converted into distances based on the sound speed inferred from the ancillary sensors, using Del Grosso’s formula^53^. The converted ranging uncertainties increase with the length of the baseline and with the uncertainties in the sound speed, at best derived from the harmonic mean of the sound speeds measured at both ends of a baseline. However, because, in 2025, we only recovered (and redeployed) stations TP7 and TP13 to access their whole datasets, all baselines shown are solely based on the sound speed at station TP7 or at station TP13 (Fig. 3 and Extended Data Fig. 3).

Two perpendicular inclinometers also monitor the beacon attitude. All tripods tilted during the seismic crisis, up to 12° for some stations (1° tilt of the 3.50-m-high tripod will cause 6 cm of displacement of the acoustic head). Because tripods are not oriented on the seafloor, we considered all possible orientations relative to the baseline direction. Given the size of the measured displacements from TP7 (Fig. 3 and Extended Data Fig. 3), the resulting uncertainties remained nearly two orders of magnitude smaller. However, for baselines from TP13, they obscured any signal (Extended Data Fig. 3b).

All tripods were blindly lowered with a cable to the seafloor from R/V Marion Dufresne. It was therefore very challenging to position them on bathymetric escarpments or cliff edges and ensure that they were stable and roughly vertical, and that each beacon could communicate with its neighbours, especially on a rugged basaltic seabed and with maps that had a pixel resolution of 20 m × 20 m at best. Deployments of the 15 beacons took 6 h per tripod on average (ranging from 3 h to 11 h).

### Bottom-pressure recorder

Vertical displacements of the seafloor were estimated with pressure sensors. A self-calibrating (that is, drift-controlled) BPR was set on the floor of the axial valley to precisely monitor its vertical deformation (cyan hexagon in Fig. 1b). For redundancy, the BPR, manufactured by RBR Ltd., has two Paroscientific Digiquartz sensors (model 46K-313) measuring the ambient ocean pressure through a single oil capillary. They also regularly measure the pressure inside the instrument casing as a calibrating ‘zero’. These ‘zero’ measurements, corrected for air-pressure variations inside the instrument with a Paroscientific barometer (model 216B-102), are used to estimate the sensor drift, with the assumption that the drift of Paroscientific pressure sensors at low pressure (that is, atmospheric pressure) is the same as that at seafloor depth, which amounts to neglecting the scale factor. Lower-resolution pressure sensors (without drift control) were also fitted to the acoustic beacons.

The BPR was lowered from the ship with a cable and dropped 30 m above the seafloor. Its recordings covered an 11-month period, from 26 February 2024 to 23 January 2025, with a sampling rate of 1 s. Calibration measurements were initially set every 2 days until 3 March, then every 7 days until 7 April, every two weeks until 6 June and finally every month. Owing to the battery running low, the last successful calibration took place in August 2024 but sampling continued. Switching the valve from ambient pressure to zero and back again consumes the most power.

Following previous work^54^, the Paroscientific raw pressure measurements were corrected for the instrumental drift derived from fitting the ‘zero’ measurements to an exponential and then a linear decay model. The contribution of tides to pressure variations was then removed using harmonic analysis (UTide software^55^) to obtain a time series of pressure reflecting, in the first order, the contribution of vertical seafloor displacement and oceanic variations unrelated to tides (Fig. 2b and Extended Data Fig. 2).

### Swath bathymetry

During the deployment cruise in February 2024 and the maintenance cruise in February 2025, we performed swath-bathymetry surveys from the hull-mounted EM 122 Kongsberg echosounder of R/V Marion Dufresne. To increase the sounding density, several overlapping swaths were collected at a speed of 5 knots. After editing and merging all tracks, DTMs with a resolution of 20 m could be achieved with a vertical uncertainty up to 5 m (about 0.2% of the water depth according to Kongsberg). These DTMs were the only information available to deploy the acoustic beacons and BPR. They are also the basis for the depth changes we documented between the two surveys in Fig. 3 and Extended Data Fig. 6.

### Elastic dislocation models

The first three large-magnitude GCMT earthquakes in the normal-faulting swarm (Mw = 4.9, 5.1 and 5.0; event numbers 1, 2 and 3 in Fig. 1b and Extended Data Table 1) coincide with sudden increases in the BPR-recorded seafloor pressure, which we interpret as coseismic seafloor subsidence by 1.7, 1.8 and 3.3 cm, respectively. This implies that these earthquakes cannot have occurred at too great a depth, otherwise they would have caused no resolvable seafloor subsidence. To quantify this, we modelled the events as uniform slip *S* on circular shear cracks of radius *R* (ref. ^38^), with a typical stress drop Δ*σ* = 3 MPa. In this framework, the average fault slip is $S=\frac{16}{7{\rm{\pi }}}\frac{\Delta \sigma }{G}R$ (in which *G* = 30 GPa denotes the shear modulus) and its radius can be estimated from the moment of each event *M*_0_ = *G*π*R*^2^*S*. We assign to each modelled rupture patch the dip of the steepest focal plane from the event focal mechanisms, 59°, 54° and 54°, respectively. This yields rupture-patch radii of 1.6, 2.0 and 1.8 km and downdip slips of 12, 15 and 13 cm. We then mesh each circular patch with triangular dislocations and use the analytical solutions of Nikkhoo and Walter^56^ to evaluate the maximum amount of seafloor subsidence that could be caused by each of these earthquakes, assuming that they occurred at depth *D* within an elastic half-space of Poisson’s ratio equal to 0.25. This amount scales as *D*^−2^ and our results (Extended Data Fig. 1) indicate that these earthquakes could not have caused seafloor subsidence greater than 1.7 cm if they had occurred any deeper than about 5 km. The Mw = 4.9 and 5.1 earthquakes, in particular, cannot have occurred at depths greater than about 3.5 and about 2.9 km, respectively. The observed coseismic subsidence thus implies that the earliest earthquakes in the swarm are confined to markedly shallow depths <5 km below the seafloor.

To model and interpret the observed seafloor subsidence and changes in acoustic baselines in terms of simple tectonomagmatic deformation sources, we used analytical models of shear and tensile dislocations in a 2D elastic half-space. We specifically considered: (1) uniform normal slip on a 60°-dipping fault, from the seafloor down to a depth *H*_f_ (ref. ^57^); (2) uniform opening of a vertical dyke between depth *H*_d1_ and *H*_d2_, located at a distance *x*_d_ to the southwest of TP7; and (3) uniform closing of a horizontal sill of cross-axis extent *W*, located at depth *H*_d2_ (ref. ^58^). The only imposed geometrical constraints were to align the fault with a southwest wall of the axial valley between TP7 and TP9, to place the dyke between TP7 and TP9 and to centre the sill at the location of TP7 with a width of 2.5 km, comparable with axial melt lenses imaged at intermediate-spreading MORs^59^. The corresponding 2D fields of (small) displacements were first recast as horizontal and vertical displacements (respectively noted as Δ*u*_*x*_ and Δ*u*_*z*_) relative to transponder TP7, set at position *x* = 0 and *z* = 0. We then converted these displacements to relative changes in slope distance between TP7 and any point located at position *x* and elevation *z* relative to TP7 (with the approximation that acoustic ray paths are straight lines).

Introducing $L=\sqrt{{x}^{2}+{z}^{2}}$, the initial slope distance between a point and TP7, the relative baseline change is:

$$\Delta L=(\sqrt{{L}^{2}+C}-L)\,{\rm{with}}\,C=\Delta {u}_{x}\times (2x+\Delta {u}_{x})+\Delta {u}_{z}\times (2z+\Delta {u}_{z}).$$

We ran 10 million random models that uniformly sample *x*_d_, *H*_d1_, *H*_d2_, *H*_f_ (<*H*_d2_) and the amount of fault slip, dyke opening and sill compaction within the bounds shown in Extended Data Fig. 5 (prior distributions as blue histograms). For each model, we calculated the RMS misfit relative to the baseline changes between transponders TP7 and TP9 (+1.13 m ± 0.26 m), TP5 (−0.51 m ± 0.19 m) and TP3 (−0.86 m ± 0.48 m), as well as the seafloor subsidence recorded at TP7 (−3.75 ± 0.55 m). We retained 2,203 models that had a RMS misfit better than 20 cm and plotted the distributions of their input parameters in Extended Data Fig. 5 (brown histograms). Bracketed values given in the text for the different parameters correspond to the 16th and 84th percentiles of these distributions, equivalent to ±1*σ* in a purely Gaussian distribution.

### Coulomb stress changes

We calculated Coulomb static stress changes caused by a segment-scale dyke intrusion event, approximated as a vertical ellipse with a vertical minor axis of 2 km and a horizontal major axis of 18 km, with an azimuth of N319. The centre of the ellipse is located 1 km below the free surface of an elastic half-space with Poisson’s ratio and shear modulus equal to 0.25 and 10 GPa, respectively. The ellipse is meshed with triangular dislocations^56^, each assigned a uniform opening of 1 m. Coulomb stress changes^60^ are computed on two types of receiver fault. The first is a strike-slip fault oriented N024, which corresponds to the focal mechanism of the Mw = 5.3 (at 22:07 on 26 April 2024) event that occurred within the Boomerang transform boundary. The second is a vertical, left-lateral strike-slip fault oriented N049 representing the Amsterdam TF orientation. Extended Data Fig. 8 shows the corresponding stress changes along horizontal cross-sections 5 km below the seafloor. We chose this depth to be below the reach of the modelled dyke to account for the fact that the seismogenic lithosphere is probably thicker around the Amsterdam and Boomerang TFs than along the SEIR segment I1.

## Online content

Any methods, additional references, Nature Portfolio reporting summaries, source data, extended data, supplementary information, acknowledgements, peer review information; details of author contributions and competing interests; and statements of data and code availability are available at 10.1038/s41586-026-10785-0.

## Supplementary information

Peer Review File

## Figures and Tables

**Extended Data Table 1:**
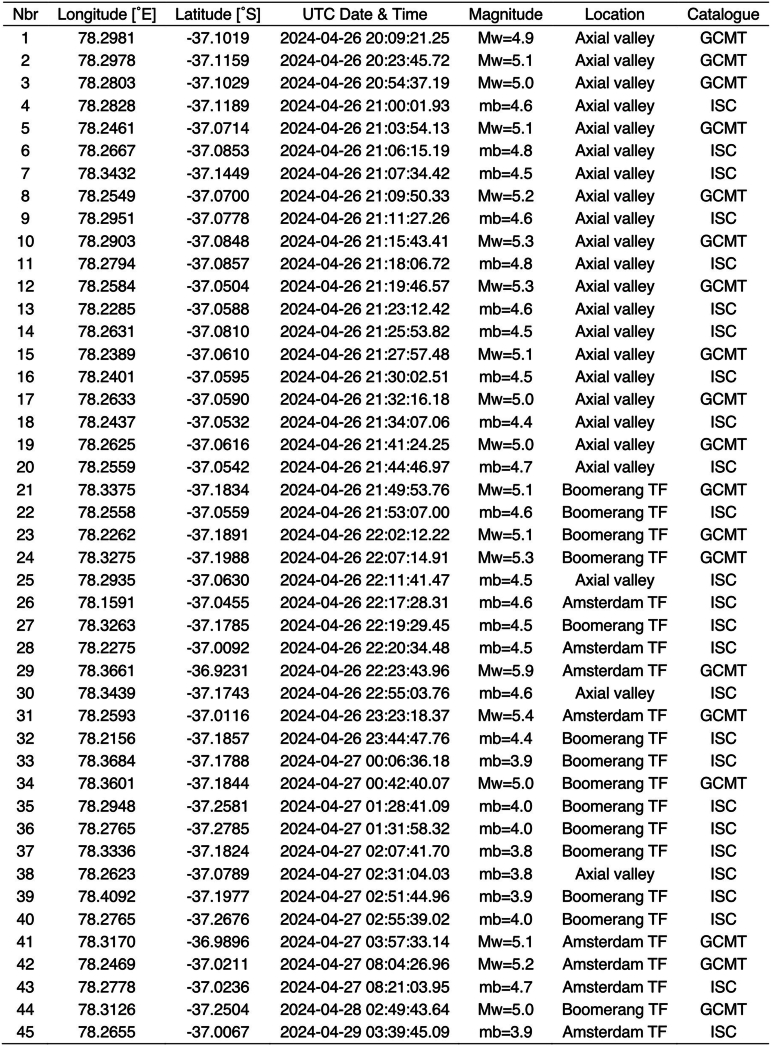
Chronology and locations of the large seismic events (as numbered in Fig. 1b)

All events were relocated from the hydroacoustic data. Magnitudes Mw and mb from the GCMT and ISC catalogues, respectively.

## Data Availability

The GCMT and ISC earthquake catalogues are publicly available. Catalogues of the hydroacoustic events reported in this study, including the relocated GCMT and ISC events, are posted in the Seanoe repository (https://www.seanoe.org/, 10.17882/115632), along with the geodetic data and bathymetric grids. Raw data will be accessible on completion of the OHA-GEODAMS project (2024–2027), according to the mandatory Data Management Plan of the MaTISS funding grant (ANR-24-CE49-7271): •Raw swath-bathymetry data are archived at the SeaDataNet repository (https://cdi.seadatanet.org/) of the French Oceanographic Fleet. Processed data will be made available at the Sextant marine data repository (https://sextant.ifremer.fr; a current compilation is available at 10.12770/342b2892-b0de-4566-bd77-520fb3bf4eaa). •Bottom-pressure recorder and hydrophones raw data will be delivered to the EPOS-France marine A-node (https://smm.epos-france.fr/). EPOS-France is a subsidiary of the European EPOS infrastructure.
