# Supplementary material for: Anatomy of a seafloor spreading event captured by in situ seismogeodesy
Source: Nature. 2026 Jul 8;655(8123):655–62. doi: 10.1038/s41586-026-10785-0 (PMC13372697; doi:10.1038/s41586-026-10785-0)
Supplement: Supplementary file 1 — Peer Review File [file 41586_2026_10785_MOESM1_ESM.pdf]

nature portfolio

Peer Review File

Anatomy of a seafloor spreading event captured by in-situ seismo-geodesy

Corresponding Author: Dr Jean-Yves Royer

Version 0:

Reviewer comments:

Referee #1

(Remarks to the Author)
Seafloor spreading is Earth's fundamental and first-order mode of shaping the ridged surface of our planet by the interplay of magmatic and tectonic processes, forming new seafloor and crust along mid-ocean ridges. Yet, this process happens on the ocean floor and hence several kilometres below the sea-surface and thousands of kilometres away from most continents, hampering scientist to follow its course. Therefore, even today it is a highly debated issue. On continentals, it is now a standard to use GNSS/GPS and satellite imagery to reveal plate motion and surface deformation associated with large earthquakes or creep. Unfortunately, the seafloor is shielded by the water of the oceans, absorbing radio and radar signals. Hence, direct measurements of plate motion or surface deformation associated with such events is hard to observe as the ocean blocks GPS signals and prevents that satellite interferometry can provide high-resolution features of the seabed. Consequently, processes well recorded on land are still poorly understood in oceanic settings. Furthermore, shiphull-mounted seafloor mapping systems have a too poor resolution to record millimetre- or centimetre-scale seafloor deformation. However, in recent years marine engineers developed new tools to measure seafloor deformation by placing devices on the ocean floor that communicate acoustically with each other, thereby obtaining direct path ranging measurements with a resolution of a few millimetres, which should allow to reveal plate motion. In addition, high resolution pressure measurements can provide evidence for minor changes in seafloor elevation and hence can monitor uplift or subsidence, especially for event-like changes (slow long-term features still might be masked by a drift of sensors). In this study, the authors have operated a network of modern sensors at a mid-ocean ridge segment of the Southeast Indian Ridge in the vicinity of the Island of Amsterdam in the Indian Ocean. By chance the network was install four months before it captured a seafloor spreading event and revealed both ~4 m of seafloor subsidence and >1 m of horizontal extension. Before, the very same segment was quite (based on global earthquake data) for decades. The observed deformation represents the deflation of a magma reservoir feeding propagating dykes being accompanied by both aseismic and seismic extension. Later, ~150 m3 of magma poured onto the seafloor, causing significant depth changes of several metres to tens of measures which could even be captured in ship-board bathymetry. A very unique and thrilling observation of a rare seafloor spreading event.

The data are unique and for the first time a spreading event on the ocean floor was captured, revealing its feature and a cascade of events following the onset of diking. In the past, at mid-ocean ridges only volcanic activity was found (events at the northern EPR and at axial volcano at the Juan de Fuca Ridge), but the associated deformation patterns remained elusive. In this case, unique observational technology was operated to obtain deformation patterns and at the same time hydroacoustic monitoring of earthquakes was used to assess the relation between magmatism, tectonics and earthquakes. In addition, observation where used in a numerical simulation to reveal its dynamics, provide an understanding of the underlying processes and setup.

I strongly support the publication of these findings. The manuscript is well structure and written and figures illustrate observations and findings very well. The methodology and data are state-of-the art and the interpretation is sound and supported by modelling. The conclusions drawn by the authors are robustness, validity, and perhaps except in the last part of the manuscript reliability. Therefore, I just have a few minor comments and therefore recommend minor revisions.

However, the last part of the paper on the “seismic moment budget” I complete fail to understand (lines 187-205). The authors fail to give any scientific justification for the calculation of budgets and fail to explain what we gain from relating the seismic moment release occurring along adjacent transform faults to the moment caused by the spreading event. It is know for a very long time that world-wide the total moment release along oceanic transform faults exceeds the total moment release of mid-ocean ridges (Frohlich & Wetzel, 2007) and even the maximum size of a mid-ocean ridges earthquakes rarely exceeds Mw~6 while oceanic transform fault may show magnitudes of Mw>7 (see Frohlich & Wetzel (2007) or Bergman & Solomon (1988) and search www.globalcmt.org). Please note that the cumulative length of active mid-ocean ridge segments is much longer than the cumulative length of transform faults. Therefore, the seismic budget of transforms versus mid-ocean

ridges will never be balanced. Actually, why should it? We know for a very long time that transform fault show a large slip deficit (e.g., Brune, 1968; Boettcher & Jordan, 2004), suggesting that transforms are dominated either by creep or slow slip, releasing perhaps as much as 85% of the geodetic slip aseismically. Consequently, any assessment of the seismic budget of transform faults is rather tenuous as they are segmented and different faults may show a very different behaviour. The same holds for mid-ocean ridge segments as we do not know how much of the plate motion and tectonic budget might be taken up by aseismic processes. Thus, I'm afraid that I cannot see the scientific reasoning behind this exercise and I cannot see that the paper can benefit from its discussion. The only measure that should add up over geological time scales is the plate separation rate (speed of the tectonic plates). However, even if possible to measure, it may take decades to centuries to obtain a full cycle. I would suggest to condense much of this section to the observation that the spreading event triggered seismic activity at an adjacent transform fault (lines 177-186), but avoid making unconstraint comments on seismic budgets.

Minor comments:

Line 52: the authors give a location uncertainty of 1-2 km for hydroacoustically located earthquakes. That's far too optimistic and none of the cited papers provides any benchmark on it. If papers discuss it, uncertainties seem to be in the order of 10 km or event larger. Furthermore, please do not ignore that there isn't even a generally agreement where T-wave are excited or where they enter into the SOFAR channel (see Wiliams et al., 2007). Please remove the number or give an appropriate reference where it was actually shown.

Throughout the manuscript, moment magnitudes are given such as 4.92 (as in line 52). That's a mathematical value calculated by an agency, but not a very robust number, ignoring any uncertainty. Please round to 4.9 or as such. The paper won't change, but magnitudes will look less awkward.

Line 91: ~4 km - measured below the seafloor or below sea level?

Line 93: basically the same: is GCMT centroid depth given below sea level or sea floor? It's difficult to compare those depth values. Please add if it's below seafloor or sea level to avoid any misunderstanding.

Line 143-144: the comparison to a fault slip derived from the Well & Coppersmith relationship is rather awkward. It either needs more details or should be removed. It might be rather unclear to most readers that we are talking about an empirically derived value for fault slip which is based on a certain model assumption and not a measured quantity. Furthermore, it's only valid for continental faults.

Line 167: What are H-wave events? They are defined in the methods, though, but show up for the first time here. Explain briefly as the term isn't well established in the geoscience community and is mainly used by a small group of scientists, including some of the authors, and hence is a kind of "slang". Just reordering the sentence may do – like "The hydrophone record at 23:22 short impulsive events, interpreted to result from lave-seawater interactions (...) which we call H-waves".

Line 178: "not parallel to the axial valley" - You mean fault planes striking obliquely to the strike of the median rift? Please rephrase and give the orientation – "not parallel" is rather puzzling and allows a wide range of different explanations.

Line 181: "straddling" is a rather funny term for Earth sciences – better rephrase.

References

Bergman, E.A. and S.C. Solomon (1988), Transform fault earthquakes in the North Atlantic: Source mechanisms and depth of faulting, J. Geophys. Res., 93, 9027-9057, doi:10.1029/JB093iB08p09027

Boettcher, M., and T. Jordan (2004), Earthquake scaling relations for mid-ocean ridge transform faults, J. Geophys. Res., 109, B12302, doi:10.1029/2004JB003110.

Brune, J. (1968), Seismic moment, seismicity, and rate of slip along major fault zones, J. Geophys. Res., 73, 777–784, doi:10.1029/JB073i002p00777.

Frohlich, C., and L.R. Wetzel (2007), Comparison of seismic moment release rates along different types of plate boundaries, Geophys. J. Int., 171, 909–920, doi:10.1111/j.1365-246X.2007.03550.x

Williams, C. M., R. A. Stephen, and D. K. Smith (2006), Hydroacoustic events located at the intersection of the Atlantis (30°N) and Kane (23°40'N) Transform Faults with the Mid-Atlantic Ridge, Geochem. Geophys. Geosyst., 7, Q06015, doi:10.1029/2005GC001127.

Well done! Ingo Grevemeyer

Referee #2

(Remarks to the Author)
Review by William Chadwick

General Comments
This manuscript presents an extraordinary suite of complementary multi-disciplinary observations that captured a seafloor spreading event and eruption in amazing detail on a segment of the Southeast Indian Ridge (SEIR). The unusually robust and full description of the event is the “high-reward” for deploying a relatively “high-risk” (temporary) mini-observatory of monitoring instrument at the site. Amazingly, the event occurred only 2 months into the experiment.
The network on monitoring instruments included: (1) an array of hydrophones to detect and locate earthquakes in the area (and better-locate those large enough to be detected by the global seismic network), as well as impulsive events from lava-water interaction on the seafloor during the eruption, (2) two separate arrays of horizontal acoustic ranging instruments, one across the center of the spreading segment and one across one of the adjacent transform faults, both designed to detect horizontal displacements on the seafloor, (3) a self-calibrating bottom pressure recorder (and internal temperature sensor), to measure vertical movements of the seafloor on the spreading axis, and (4) repeated ship-based bathymetric mapping of the area to detect depth changes due to the eruption of lava during the event. The paper skillfully blends all these observations to tell a compelling story about how this remarkable event unfolded.
The key co-incident observations include: (1) the sequence of earthquakes, including their focal mechanisms and migrating locations along the ridge axis and into the adjacent transform faults, (2) the subsidence of the axial valley floor by ~ 4 meters, (3) the extension of acoustic ranging baselines to the west of the ridge axis and contraction of other baselines east of the axis by 0.5-1.5 meters, and (4) the location and extent of depth changes along the ridge axis, showing where lava flows were erupted and constraining their thickness and cumulative volume.
The observations allow the authors to explore possible mechanical models that can reasonably explain the observations, including a deflating magma lens (sill) beneath the center of the ridge segment, a vertical dike intruded along the ridge axis, and slip on one of the normal faults bounding the axial valley. The authors conclude that the apparent seismic moment deficit related to faulting along the ridge segment implies that much of the normal fault slip during such events is aseismic, which has broader implications for mid-ocean ridge systems worldwide.
The manuscript was a pleasure to read. The writing is clear and concise. The figures are excellent and dense with useful information. Nevertheless, I have included some suggested edits and comments in an annotated manuscript file to try to clarify points, improve the wording, and suggest additional references in a few places. I recommend publication after minor revision.
Simply put, no seafloor spreading event has ever been documented with this level of detail, and the new data reveal fascinating relationships between seismicity, deformation, magmatic intrusion and volcanism. This is an extraordinary manuscript and there is no doubt in my mind that it is deserving to be published in Nature, because the observations are unprecedented, robust, and illuminating.

Specific comments keyed to line numbers in the manuscript
• Line 15: Should “a valley bounding fault” be plural here? I think you made it singular, because your model in Figure 4 only includes one fault, but certainly all the seismicity reflects motion on multiple faults, both along the ridge segment and along the transform faults. It sounds “wrong” to only interpret slip along a singular fault.
• Lines 17-19: I think you need to rephrase this last sentence of the Abstract because it is not clear how the "unique observations" are related to "aseismic slip" without some additional explanation (otherwise this sentence seems "unsupported" from what comes before it). I have suggested a possible rewording.
• Line 37: You should definitely add this reference here, since you mention deformation AND seismicity. This paper describes the seismicity during the 2015 eruption at Axial Seamount and was a companion paper to Nooner & Chadwick, 2016:
Wilcock, W. S. D., M. Tolstoy, F. Waldhauser, C. Garcia, Y. J. Tan, D. R. Bohnenstiehl, J. Caplan-Auerbach, R. P. Dziak, A. F. Arnulf, and M. E. Mann (2016), Seismic constraints on caldera dynamics from the 2015 Axial Seamount eruption, Science, 354(6318), 1395–1399, doi:10.1126/science.aah5563.
Optional additional references include this one which describes the cabled observatory at Axial Seamount:
Kelley, D. S., J. R. Delaney, and S. K. Juniper (2014), Establishing a new era of submarine volcanic observatories: Cabling Axial Seamount and the Endeavour Segment of the Juan de Fuca Ridge, Mar. Geol., 352, 426–450, doi:10.1016/j.margeo.2014.03.010.
... and this one, which discusses deformation and seismicity at Axial Seamount since the 2015 eruption:
Chadwick, W. W., Jr., W. S. D. Wilcock, S. L. Nooner, J. W. Beeson, A. M. Sawyer, and T.-K. Lau (2022), Geodetic Monitoring at Axial Seamount Since its 2015 Eruption Reveals a Waning Magma Supply and Tightly Linked Rates of Deformation and Seismicity, Geochem. Geophys. Geosyst., 23(1), e2021GC010153, doi:10.1029/2021GC010153.
• Lines 58-59: FYI, there are examples of horizontal acoustic ranging systems from Japan too:
Osada, Y., M. Kido, and H. Fujimoto (2012), A long-term seafloor experiment using an acoustic ranging system: precise horizontal distance measurements toward detection of seafloor crustal deformation, Ocean Engineering, 51, 28–33.
Osada, Y., M. Kido, H. Fujimoto, and Y. Kaneda (2008), Development of a seafloor acoustic ranging system toward the seafloor cable network system, Ocean Engineering, 35, 1401–1405, doi:10.1016/j.oceaneng.2008.07.007.
• Line 68: I think it's important to briefly mention how BPR drift is controlled, or what type of BPR this is (a commercial or custom instrument).
• Line 70: Fox (1999) might be a better reference than Fox (1993) here, because it refers to the same event as the Dziak and Embley references, and it has a more definitive signal.
Fox, C. G. (1999), In situ ground deformation measurements from the summit of Axial Volcano during the 1998 volcanic episode, Geophys. Res. Lett., 26(23), 3437–3440.
• Line 75-76: This description is slightly confusing, because the Mw=4.92 event was not the first detected earthquake, it was the first in the GCMC catalog with a focal mechanism determination (as shown in Figure 2). The hydrophone array detected some earthquakes a few minutes before this one. So probably just need to rephrase this slightly to acknowledge this.
• Line 77: Looks like 17 hydroacoustic events in figure 2A (rather than 15).

• Lines 81-82: Could also add Dziak and Fox (1999a) here.
• Lines 87-89: This step-wise co-seismic subsidence really reminds me of the caldera collapse sequence at Kilauea volcano, Hawaii, in 2018, described in Neal et al., 2019 (although obviously a different setting):
Neal, C. A., et al. (2019), The 2018 rift eruption and summit collapse of Kīlauea Volcano, Science, 363, 367–374, doi:10.1126/science.aav7046.
• Line 119: This should be Fox (1999), rather than Fox (1993) ... but actually maybe that should just be omitted, because that paper is about vertical deformation, whereas this sentence is talking about horizontal displacements. Also, the Chadwick et al reference is maybe not a great analogy, because that horizontal ranging was not across a zone where a dike was intruded. Nevertheless, it is true that the changes measured were much smaller in that case.
• Line 125-126: Is the built-in pressure data shown somewhere?
• Line 132: Nooner & Chadwick (2016) model co-eruption subsidence during the 2015 eruption at Axial Seamount. They do consider a penny-shaped crack sill model, but their preferred deformation model is not a sill. You might consider adding this reference, which does include various sill models for the same dataset, but also considering newer AUV repeat bathymetry data:
Slead, S., M. Wei, S. L. Nooner, W. W. Chadwick Jr., D. W. Caress, and J. W. Beeson (2024), Compartmentalization of Axial Seamount's magma reservoir inferred by analytical and numerical deformation modeling with realistic geometry, Journal of Geophysical Research: Solid Earth, 129(5), e2023JB028414, doi:10.1029/2023JB028414.
• Line 152: A couple of alternative references to consider rather than Chadwick et al., 1998:
Fox, C. G., W. W. Chadwick, Jr., and R. W. Embley (1992), Detection of changes in ridge-crest morphology using repeated multibeam sonar surveys, Journal of Geophysical Research: Solid Earth, 97, 11149–11162, doi:10.1029/92JB00601.
This was the first time repeat bathymetry was done on a MOR, but it was pre-GPS navigation, so we had to have extra steps to align the bathymetric grids. The Chadwick et al. 1998 paper also had to deal with pre-GPS navigated bathymetry, so is noisier than is possible now. A post-GPS example would be this one (related to the 2015 eruption at Axial Seamount):
Chadwick, W. W., Jr., B. P. Paduan, D. A. Clague, B. M. Dreyer, S. G. Merle, A. M. Bobbitt, D. W. Caress, B. Philip, D. S. Kelley, and S. L. Nooner (2016), Voluminous eruption from a zoned magma body after an increase in supply rate at Axial Seamount, Geophys. Res. Lett., 43, 12063–12070, doi:10.1002/2016GL071327.
You could just say “grids” instead of “Digital Terrain Models”.
• Lines 155-156: To me it looks like there are (maybe) two parallel lines of depth change (lava flows) in Figure 3 (also Extended Data Figure 6). I'm curious of the authors interpret this as due to eruption from two parallel dikes (which would be unusual), or alternatively that either the flows are all connected by thinner portions that are below the detection limit, or perhaps that magma also intruded up along the normal fault at the western edge of the axial valley. I'd be curious to see the (noisier) raw difference grid; Figure 3 looks like it only shows depth differences above a 5 m threshold. Maybe that should be added as another panel in Extended Data Figure 6. The caption to Extended Data Figure 6 seems to suggest the authors favor the second of my alternative hypotheses above (magma also intruded up along the western fault). I think this interpretation should be added as a sentence or two in the main text (with appropriate acknowledgement of uncertainty) and not be buried in the caption of an Extended Data figure.
• Line 163: Could add a reference to Chadwick et al. (2016) here, since the volume of the 2015 eruption at Axial Seamount about about 150 million m3.
Chadwick, W. W., Jr., B. P. Paduan, D. A. Clague, B. M. Dreyer, S. G. Merle, A. M. Bobbitt, D. W. Caress, B. Philip, D. S. Kelley, and S. L. Nooner (2016), Voluminous eruption from a zoned magma body after an increase in supply rate at Axial Seamount, Geophys. Res. Lett., 43, 12063–12070, doi:10.1002/2016GL071327.
Clague et al. (2017) has a slightly refined volume estimate for the same eruption:
Clague, D. A., J. B. Paduan, D. W. Caress, W. W. Chadwick Jr., M. L. Saout, B. Dreyer, and R. Portner (2017), High-resolution AUV mapping and targeted ROV observations of three historical lava flows at Axial Seamount, Oceanography, 30(4), 82–99, doi:10.5670/oceanog.2017.426.
• Line 167: Need to define "H-wave" the first time you use it.
• Line 168: Lava-water interaction signals were also described in these references from the 2015 Axial Seamount eruption:
Wilcock, W. S. D., M. Tolstoy, F. Waldhauser, C. Garcia, Y. J. Tan, D. R. Bohnenstiehl, J. Caplan-Auerbach, R. P. Dziak, A. F. Arnulf, and M. E. Mann (2016), Seismic constraints on caldera dynamics from the 2015 Axial Seamount eruption, Science, 354(6318), 1395–1399, doi:10.1126/science.aah5563.
Le Saout, M., D. R. Bohnenstiehl, J. B. Paduan, and D. A. Clague (2020), Quantification of eruption dynamics on the north rift at Axial Seamount, Juan de Fuca Ridge, Geochem. Geophys. Geosyst., 21, e2020GC009136, doi:10.1029/2020GC009136.
• Line 171: This is also the interpretation from the 2015 Axial eruption - that the hydroacoustic impulsive events mark the duration of lava extrusion. (Wilcock et al., 2016; Le Saout et al., 2020). Can the impulsive hydroacoustic events be located? If so, do they cluster where the depth changes occur? What do their locations in space and time tell us about the emplacement of the lava flows [like Le Saout et al. (2020)]? Will this be part of a future study?
• Line 203: Maybe another place to point out the similarity to Kilauea's 2018 collapse (Neal et al., 2019)?
• Line 472: I added this sentence to the end of this paragraph: “ We may be able to reduce the uncertainty in the ranging measurements further after all the instruments and their recorded (temperature) data are recovered at the end of the experiment.” Is this true?
• Figure 1B: There is a lot of information in this figure, which is great, but I worry that it will not be legible if it is reduced any in size when published (the font size showing the earthquake magnitudes is already very small, for example).
• Figure 2A: Change label here from “BPR” to "BPR location".
• Figure 3: Maybe make magma lens a different color (light red?), to avoid confusion because the lava flows are grey? If so, then make the dashed line representing the model dike be a darker red, so it stands out. Also make the labels “Dike” and “Magma lens” the appropriate colors. Add black lines to the tops of the inset plots, so they are completely enclosed and separated from the underlying plot.
• Figure 4: Consider changing the label "Baseline changes" to "Horizontal range baseline changes", just to clarify. If you do that, adjust the wording in the figure caption too. In the caption, I assume you aren't really "summing up" the horizontal and

vertical displacements, but rather using them to calculate a range change or slope distance, right? This should be rephrased to clarify.

- Extended Data Figure 2: What happens after May 2? The number of impulsive events has not leveled off by then. How long does it keep increasing? Or when do the impulsive events stop?
- Extended Data Figure 4: I would change the label on the figure from "drainage" to "sill"
- Extended Data Figure 5: Also explain what the brown histograms are in the caption.

Version 1:

Reviewer comments:

Referee #1

(Remarks to the Author)
This manuscript represents a revised version of a paper I had previously reviewed. I greatly appreciate that most of my comments were appreciated and the criticism acknowledged and hence the manuscript greatly advanced – especially the discussion of the released seismic budget and its relationship to the adjacent transform faults. There are only a very few and very minor points – which I believe can also handled during final editing of the manuscript and hence after it acceptance.

Line 90: GCMT centroid depth is always given below sea level – though the authors state it is below seafloor. However, I checked the value reported in the online catalogue and the reported depth agrees which the online catalogue, hence are below sea level. Please correct.

Lines 133-145: Paragraph on dislocation modelling – the paragraph is largely written for a community dealing with modelling of similar types of problems. Thus, I do not know how many readers will understand it. For example, there might be a large number of reader who may not understand well what compaction of a sill by 10-20 m (line 136) suggests. Further, give the range for the opening (line 137) – you just say meters; however, just 1-2 m or tens of metres? The same holds for the slip (line 137, too). Please give the range – 1-2 m or more? The careful reader can deduce those from the extended figures – but it should be clear just by reading the text.

Line 139-140: Figure 4 does not show any sill that is 2500 m (i.e, 2.5 km!) wide. Actually, a 2500 m wide sill would build nearly half of the crust in just one event; rather unlikely. Please correct or describe the process more clearly.

New Figure 5: the left-hand side of the plot is “half empty” (Fig 5a). Why not cut the vertical axis at 1000 PJ/km?

Great experiment! Ingo Grevemeyer

Referee #2

(Remarks to the Author)
Review of revised manuscript by William Chadwick

I find that the authors have addressed all my earlier comments, concerns, and suggested edits. The only remaining issue I noticed was that in the caption to Figure 3, it says "(light red rectangle)" when referring to the deflating magma lens in the figure, but it should say "(purple rectangle)", because the authors changed the color of the rectangle during revision. Otherwise, I think the paper is ready to publish. I note that the authors have made some very valuable additions to the Extended Data Figures, based on my previous comments. This is a very remarkable and exciting paper!

Open Access This Peer Review File is licensed under a Creative Commons Attribution 4.0 International License, which permits use, sharing, adaptation, distribution and reproduction in any medium or format, as long as you give appropriate credit to the original author(s) and the source, provide a link to the Creative Commons license, and indicate if changes were made.
In cases where reviewers are anonymous, credit should be given to 'Anonymous Referee' and the source.
The images or other third party material in this Peer Review File are included in the article's Creative Commons license, unless indicated otherwise in a credit line to the material. If material is not included in the article's Creative Commons license and your intended use is not permitted by statutory regulation or exceeds the permitted use, you will need to obtain permission directly from the copyright holder.
To view a copy of this license, visit https://creativecommons.org/licenses/by/4.0/

Responses to referees (Ref: MS 2025-11-29673)

Referee #1:

Seafloor spreading is Earth's fundamental and first-order mode of shaping the ridged surface of our planet by the interplay of magmatic and tectonic processes, forming new seafloor and crust along mid-ocean ridges. Yet, this process happens on the ocean floor and hence several kilometres below the sea-surface and thousands of kilometres away from most continents, hampering scientist to follow its course. Therefore, even today it is a highly debated issue. On continentals, it is now a standard to use GNSS/GPS and satellite imagery to reveal plate motion and surface deformation associated with large earthquakes or creep. Unfortunately, the seafloor is shielded by the water of the oceans, absorbing radio and radar signals. Hence, direct measurements of plate motion or surface deformation associated with such events is hard to observe as the ocean blocks GPS signals and prevents that satellite interferometry can provide high-resolution features of the seabed. Consequently, processes well recorded on land are still poorly understood in oceanic settings. Furthermore, shiphull-mounted seafloor mapping systems have a too poor resolution to record millimetre- or centimetre-scale seafloor deformation. However, in recent years marine engineers developed new tools to measure seafloor deformation by placing devices on the ocean floor that communicate acoustically with each other, thereby obtaining direct path ranging measurements with a resolution of a few millimetres, which should allow to reveal plate motion. In addition, high resolution pressure measurements can provide evidence for minor changes in seafloor elevation and hence can monitor uplift or subsidence, especially for event-like changes (slow long-term features still might be masked by a drift of sensors). In this study, the authors have operated a network of modern sensors at a mid-ocean ridge segment of the Southeast Indian Ridge in the vicinity of the Island of Amsterdam in the Indian Ocean. By chance the network was install four months before it captured a seafloor spreading event and revealed both ~4 m of seafloor subsidence and >1 m of horizontal extension. Before, the very same segment was quite (based on global earthquake data) for decades. The observed deformation represents the deflation of a magma reservoir feeding propagating dykes being accompanied by both aseismic and seismic extension. Later, ~150 m3 of magma poured onto the seafloor, causing significant depth changes of several metres to tens of measures which could even be captured in ship-board bathymetry. A very unique and thrilling observation of a rare seafloor spreading event.

The data are unique and for the first time a spreading event on the ocean floor was captured, revealing its feature and a cascade of events following the onset of diking. In the past, at mid-ocean ridges only volcanic activity was found (events at the northern EPR and at axial volcano at the Juan de Fuca Ridge), but the associated deformation patterns remained elusive. In this case, unique observational technology was operated to obtain deformation patterns and at the same time hydroacoustic monitoring of earthquakes was used to assess the relation between magmatism, tectonics and earthquakes. In addition, observation were used in a numerical simulation to reveal its dynamics, provide an understanding of the underlying processes and setup.

I strongly support the publication of these findings. The manuscript is well structure and written and figures illustrate observations and findings very well. The methodology and data are state-of-the art and the interpretation is sound and supported by modelling. The conclusions drawn by the authors are robustness, validity, and perhaps except in the last part of the manuscript reliability. Therefore, I just have a few minor comments and therefore recommend minor revisions.

However, the last part of the paper on the "seismic moment budget" I complete fail to understand (lines 187-205). The authors fail to give any scientific justification for the calculation of budgets and fail to explain what we gain from relating the seismic moment release occurring along adjacent transform faults to the moment caused by the spreading event. It is known for a very long time that world-wide the total moment release along oceanic transform faults exceeds the total moment release of mid-ocean ridges (Frohlich & Wetzel, 2007) and even the maximum size of a mid-ocean ridges earthquakes rarely exceeds Mw~6 while oceanic transform fault may show magnitudes of Mw>7 (see Frohlich & Wetzel (2007) or Bergman & Solomon (1988) and search www.globalcmt.org). Please note that the cumulative length of active mid-ocean ridge segments is much longer than the cumulative length of transform faults. Therefore, the seismic budget of transforms versus mid-ocean ridges will never be balanced. Actually, why should it? We know for a very long time that transform fault show a large slip deficit (e.g., Brune, 1968; Boettcher & Jordan, 2004), suggesting that transforms are dominated either by creep or slow slip, releasing perhaps as much as 85% of the geodetic slip aseismically. Consequently,

any assessment of the seismic budget of transform faults is rather tenuous as they are segmented and different faults may show a very different behaviour. The same holds for mid-ocean ridge segments as we do not know how much of the plate motion and tectonic budget might be taken up by aseismic processes. Thus, I'm afraid that I cannot see the scientific reasoning behind this exercise and I cannot see that the paper can benefit from its discussion. The only measure that should add up over geological time scales is the plate separation rate (speed of the tectonic plates). However, even if possible to measure, it may take decades to centuries to obtain a full cycle. I would suggest to condense much of this section to the observation that the spreading event triggered seismic activity at an adjacent transform fault (lines 177-186), but avoid making unconstraint comments on seismic budgets.

We believe ending the paper by discussing the 2024 event in the context of decadal-scale activity on segment I1 and abutting transforms is a worthy exercise that directly addresses some of the key questions raised in the introduction (e.g., "*Many questions remain regarding how stresses accumulate between these events*[5,6] *(Chadwell & Spiess, 2008; Olive & Dublanchet, 2020), the degree to which activity on spreading segments and nearby TFs is synchronized*[7] *(Dziak et al. 2009), and why MORs release so little seismic energy given their rapid time-averaged deformation rates*[8,9]") and in the referee's comment (highlighted above). That said, we acknowledge that the final part of the initial manuscript had some ambiguous phrasing suggesting that moment release on the spreading segment should somehow catch up with the moment release of the abutting transforms. We agree that this is not the case and have thoroughly revised the last paragraphs, and accompanying Fig. 5, to clarify our message, in line with the reviewer's comments.

First: we have removed any mention (and calculation) of expected moment rates on the transforms, as the state of coupling on transforms is indeed beyond the scope of our study. We still plot the normalized moment release on the transforms in Fig. 5, but only to highlight what we believe to be an interesting observation: that while the spreading event clearly triggered activity on the transforms, the associated seismic energy release does not at all "stand out" compared to the average moment rate documented by the GCMT catalog over the last few decades. It also visually illustrates how seismically quiescent the I1 segment has been compared to the TFs over that same time span.

After pointing this out, the remainder of the paper focuses on balancing the moment budget of the I1 segment, independently of the transforms. We specifically discuss the balance of geometric moment (or horizontal potency, as defined for example by Ben Zion (2003), which amounts to a balance of horizontal strain) leading up to and during the 2024 spreading event. We do so by recognizing that our elastic models provide a distribution of plausible faulting moments, but also of diking "moments", which together amount to a horizontal potency of 12.3×10^3 m^3/meter along axis: the equivalent of 39 years of extension on a 5 km-thick axial lithosphere at 63 mm/yr. This provides a reasonable time scale of recurrence for the observed event. It also shows that the 2024 magmatic intrusion accounted for ~90% of that extension, and that the remaining ~10% (tectonic) was ~25% seismic and ~75% aseismic. This is the first time that these various components can be evaluated in a seafloor spreading event. Though these numbers are associated with large error bars (which we assess systematically using the models' pdfs), they add crucial new information to the moment deficit debate, and as such are a pertinent way to conclude the paper. We also note that referee #2 expressed little concern about this part, except for its overly concise wording in the summary, which we have revised accordingly.

References:
Ben-Zion, Y. (2003). Appendix 2 - Key Formulas in Earthquake Seismology. In W. H. K. Lee, H. Kanamori, P. C. Jennings, & C. Kisslinger (Eds.), *International Geophysics* (Vol. 81, pp. 1857–1875). Academic Press. https://doi.org/10.1016/S0074-6142(03)80304-2

Minor comments:

Line 52: the authors give a location uncertainty of 1-2 km for hydroacoustically located earthquakes. That's far too optimistic and none of the cited papers provides any benchmark on it. If papers discuss it, uncertainties seem to be in the order of 10 km or event larger. Furthermore, please do not ignore that there isn't even a generally agreement where T-wave are excited or where they enter into the SOFAR channel (see Williams et al., 2007). Please remove the number or give an appropriate reference where it was actually shown.

Hydroacoustically-determined earthquake locations in MOR settings are notoriously difficult to benchmark due to the lack of ground-truth. Fox et al. (2001) used the Loihi eruption, off the Big Island of Hawaii, as a known source location to validate the analytical 1-2 km estimation. Slack et al. (1999; added in the references) found similar uncertainties using a Monte Carlo approach. Most seismicity studies based on wide hydroacoustic networks (e.g. ref. 7, 18, 19; Bohnenstiehl et al., 2002; Smith et al., 2003; …), including Williams et al. (2006), which the reviewer refers to, report lat and lon errors mostly in the range of 0.01 to 0.02 degrees (= 1 to 2 km). Pan and Dziewonski (2005) compared hydroacoustic (from autonomous hydrophones) and land-based catalogs, and considered the AUH locations as more accurate.

Following the approach designed at PMEL (Fox et al., 2001), with 4 up to 6-7 arrival times, one can derive the uncertainties in latitude, longitude and origin time from the covariance matrix of the non-linear least-square inversion. In this paper, they do not exceed 2 km (major axis) vs 1 km (minor axis) at 1σ (68% confidence level); in addition, similarly to Slack et al. (1999), for each event, we systematically performed 10,000 random Monte Carlo draws within a one-second interval from the arrival times and obtained the same figures. This is now explicitly mentioned in the Methods section. For shallow earthquakes, particularly in a spreading segment with a smooth bathymetry, 1-2 km is a fair estimate of the location uncertainties of its epicenter (i.e. acoustic radiator) **inside the hydrophone array**. The scattering in the AUH event locations is due to the difficulty to distinguish low-magnitude events closely spaced in time (see Extended Data Figure 7); still, the majority of them focuses in the axial valley. Furthermore, the alignment of the main events (ISC, GCMT) in the 2km-wide valley is quite remarkable, as those along the transforms. These geological arguments validate Fox et al. (2001) and others' estimates.

References:

Bohnenstiehl, D. R., Tolstoy, M., Dziak, R. P., Fox, C. G., & Smith, D. K. (2002). Aftershock sequences in the mid-ocean ridge environment: an analysis using hydroacoustic data. *Tectonophysics*, *354*(1), 49–70. https://doi.org/10.1016/S0040-1951(02)00289-5

Pan, J. F., & Dziewonski, A. M. (2005). Comparison of mid-oceanic earthquake epicentral differences of travel time, centroid locations, and those determined by autonomous underwater hydrophone arrays. *Journal of Geophysical Research*, *110*(B7), B07302. https://doi.org/10.1029/2003JB002785

Slack, P. D., Fox, C. G., & Dziak, R. P. (1999). P wave detection thresholds, Pn velocity estimates, and T wave location uncertainty from oceanic hydrophones. *Journal of Geophysical Research*, *104*(B6), 13061–13072. https://doi.org/10.1029/1999JB900112

Smith, D. K., Escartin, J., Cannat, M., Tolstoy, M., Fox, C. G., Bohnenstiehl, D. R., & Bazin, S. (2003). Spatial and temporal distribution of seismicity along the northern Mid-Atlantic Ridge. *Journal of Geophysical Research*, *108*(B3), 2167. https://doi.org/10.1029/2002JB001964

Throughout the manuscript, moment magnitudes are given such as 4.92 (as in line 52). That's a mathematical value calculated by an agency, but not a very robust number, ignoring any uncertainty. Please round to 4.9 or as such. The paper won't change, but magnitudes will look less awkward.

The Mw moment-magnitudes were recalculated from the moment tensor components provided by GCMT. Nonetheless, we agree with the reviewer. Our main reason for keeping 2 decimals in Mw was to distinguish them from mb body-wave magnitude in the figures. We have now removed the extra decimal and, in the figures, outlined in black the Mw's and in blue the mb's.

Line 91: ~4 km - measured below the seafloor or below sea level?

Below seafloor. Added in the text.

Line 93: basically the same: is GCMT centroid depth given below sea level or sea floor? It's difficult to compare those depth values. Please add if it's below seafloor or sea level to avoid any misunderstanding.

GCMT provides centroid depths below sea-level. Line 90, we subtracted a water depth of 2 km to the centroid depths given by GCMT for consistency with that calculated two lines above below seafloor (5 km).

Line 143-144: the comparison to a fault slip derived from the Well & Coppersmith relationship is rather awkward. It either needs more details or should be removed. It might be rather unclear to most readers that we are talking about an empirically derived value for fault slip which is based on a certain model assumption and not a measured quantity. Furthermore, it's only valid for continental faults.

This comment prompted us to re-evaluate our estimates of fault slip associated with the normal faulting earthquakes: instead of using the empirical scaling of Wells and Coppersmith, which are indeed built on a compilation of continental ruptures, we now rely on the classical circular crack model of Brune (1970) with a typical stress drop of 3 MPa. We have revised the corresponding section of the Methods to explain this in detail, and now refer to the Methods in the sentence pointed out by the reviewer. This should make it clear that we are talking about model-derived coseismic displacements and not a direct observation. That said, the comparison is meant in an order-of-magnitude sense, for which classical (e.g., constant stress drop) scalings all agree the slip associated with a Mw5 should be on the order of ~10 cm, one order of magnitude less than the metric slip we document with geodesy.

Reference:

Brune, J. N. (1970). Tectonic stress and the spectra of seismic shear waves from earthquakes. Journal of Geophysical Research (1896-1977), 75(26), 4997–5009. https://doi.org/10.1029/JB075i026p04997

Line 167: What are H-wave events? They are defined in the methods, though, but show up for the first time here. Explain briefly as the term isn't well established in the geoscience community and is mainly used by a small group of scientists, including some of the authors, and hence is a kind of "slang". Just reordering the sentence may do – like "The hydrophone record at 23:22 short impulsive events, interpreted to result from lave-seawater interactions (...) which we call H-waves".

Explanation is now given in the main text: "*the occurrence of numerous short (< 10s) and energetic hydroacoustic events, called H-waves and generated in the water-column by lava-seawater interactions*[10,44–47]*(Wilcock et al., 2016; Tan et al. 2016; Le Saout et al., 2020; Bazin et al. 2022; Wang et al. 2025)*"

Line 178: "not parallel to the axial valley" - You mean fault planes striking obliquely to the strike of the median rift? Please rephrase and give the orientation – "not parallel" is rather puzzling and allows a wide range of different explanations.

Fixed, thank you.

Line 181: "straddling" is a rather funny term for Earth sciences – better rephrase.

Funny as it may sound, we believe the term is appropriate. It appears, for example, in the 1st sentence of section 4 in Williams et al. (2006), mentioned by the reviewer, à propos a hydrophone network that "*straddles the MAR from 35°N–15°N*".

References

Bergman, E.A. and S.C. Solomon (1988), Transform fault earthquakes in the North Atlantic: Source mechanisms and depth of faulting, J. Geophys. Res., 93, 9027-9057, doi:10.1029/JB093iB08p09027

Boettcher, M., and T. Jordan (2004), Earthquake scaling relations for mid-ocean ridge transform faults, J. Geophys. Res., 109, B12302, doi:10.1029/2004JB003110.

Brune, J. (1968), Seismic moment, seismicity, and rate of slip along major fault zones, J. Geophys. Res., 73, 777–784, doi:10.1029/JB073i002p00777.

Frohlich, C., and L.R. Wetzel (2007), Comparison of seismic moment release rates along different types of plate boundaries, Geophys. J. Int., 171, 909–920, doi:10.1111/j.1365-246X.2007.03550.x

Williams, C. M., R. A. Stephen, and D. K. Smith (2006), Hydroacoustic events located at the intersection of the Atlantis (30°N) and Kane (23°40'N) Transform Faults with the Mid-Atlantic Ridge, Geochem. Geophys. Geosyst., 7, Q06015, doi:10.1029/2005GC001127.

Well done! Ingo Grevemeyer

We thank Dr. Grevemeyer for his comments that have helped strengthen the manuscript.

****

Referee #2:

Review by William Chadwick

General Comments

This manuscript presents an extraordinary suite of complementary multi-disciplinary observations that captured a seafloor spreading event and eruption in amazing detail on a segment of the Southeast Indian Ridge (SEIR). The unusually robust and full description of the event is the "high-reward" for deploying a relatively "high-risk" (temporary) mini-observatory of monitoring instrument at the site. Amazingly, the event occurred only 2 months into the experiment.

The network on monitoring instruments included: (1) an array of hydrophones to detect and locate earthquakes in the area (and better-locate those large enough to be detected by the global seismic network), as well as impulsive events from lava-water interaction on the seafloor during the eruption, (2) two separate arrays of horizontal acoustic ranging instruments, one across the center of the spreading segment and one across one of the adjacent transform faults, both designed to detect horizontal displacements on the seafloor, (3) a self-calibrating bottom pressure recorder (and internal temperature sensor), to measure vertical movements of the seafloor on the spreading axis, and (4) repeated ship-based bathymetric mapping of the area to detect depth changes due to the eruption of lava during the event. The paper skillfully blends all these observations to tell a compelling story about how this remarkable event unfolded.

The key co-incident observations include: (1) the sequence of earthquakes, including their focal mechanisms and migrating locations along the ridge axis and into the adjacent transform faults, (2) the subsidence of the axial valley floor by ~ 4 meters, (3) the extension of acoustic ranging baselines to the west of the ridge axis and contraction of other baselines east of the axis by 0.5-1.5 meters, and (4) the location and extent of depth changes along the ridge axis, showing where lava flows were erupted and constraining their thickness and cumulative volume.

The observations allow the authors to explore possible mechanical models that can reasonably explain the observations, including a deflating magma lens (sill) beneath the center of the ridge segment, a vertical dike intruded along the ridge axis, and slip on one of the normal faults bounding the axial valley. The authors conclude that the apparent seismic moment deficit related to faulting along the ridge segment implies that much of the normal fault slip during such events is aseismic, which has broader implications for mid-ocean ridge systems worldwide.

The manuscript was a pleasure to read. The writing is clear and concise. The figures are excellent and dense with useful information. Nevertheless, I have included some suggested edits and comments in an annotated manuscript file to try to clarify points, improve the wording, and suggest additional references in a few places. I recommend publication after minor revision.

Simply put, no seafloor spreading event has ever been documented with this level of detail, and the new data reveal fascinating relationships between seismicity, deformation, magmatic intrusion and volcanism. This is an extraordinary manuscript and there is no doubt in my mind that it is deserving to be published in Nature, because the observations are unprecedented, robust, and illuminating.

We thank Dr. Chadwick for his enthusiastic and helpful feedback.

Specific comments keyed to line numbers in the manuscript

We also addressed the minor comments in the annotated manuscript, particularly the suggestions to improve the figures, which are spelled out further below.

• Line 15: Should "a valley bounding fault" be plural here? I think you made it singular, because your model in Figure 4 only includes one fault, but certainly all the seismicity reflects motion on multiple faults, both along the ridge segment and along the transform faults. It sounds "wrong" to only interpret slip along a singular fault.

Indeed, fixed.

• Lines 17-19: I think you need to rephrase this last sentence of the Abstract because it is not clear how the "unique observations" are related to "aseismic slip" without some additional explanation (otherwise this sentence seems "unsupported" from what comes before it). I have suggested a possible rewording.

We agree; we found an alternative that reflects the revised final paragraphs of the manuscript, following the comments of Reviewer 1.

• Line 37: You should definitely add this reference here, since you mention deformation AND seismicity. This paper describes the seismicity during the 2015 eruption at Axial Seamount and was a companion paper to Nooner & Chadwick, 2016:

Wilcock, W. S. D., M. Tolstoy, F. Waldhauser, C. Garcia, Y. J. Tan, D. R. Bohnenstiehl, J. Caplan-Auerbach, R. P. Dziak, A. F. Arnulf, and M. E. Mann (2016), Seismic constraints on caldera dynamics from the 2015 Axial Seamount eruption, Science, 354(6318), 1395–1399, doi:10.1126/science.aah5563.

Optional additional references include this one which describes the cabled observatory at Axial Seamount:

Kelley, D. S., J. R. Delaney, and S. K. Juniper (2014), Establishing a new era of submarine volcanic observatories: Cabling Axial Seamount and the Endeavour Segment of the Juan de Fuca Ridge, Mar. Geol., 352, 426–450, doi:10.1016/j.margeo.2014.03.010.

... and this one, which discusses deformation and seismicity at Axial Seamount since the 2015 eruption:

Chadwick, W. W., Jr., W. S. D. Wilcock, S. L. Nooner, J. W. Beeson, A. M. Sawyer, and T.-K. Lau (2022), Geodetic Monitoring at Axial Seamount Since its 2015 Eruption Reveals a Waning Magma Supply and Tightly Linked Rates of Deformation and Seismicity, Geochem. Geophys. Geosyst., 23(1), e2021GC010153, doi:10.1029/2021GC010153.

We thank the reviewer for pointing out these additional references. Referring to Wilcock et al. (2016) is fully pertinent.

• Lines 58-59: FYI, there are examples of horizontal acoustic ranging systems from Japan too:

Osada, Y., M. Kido, and H. Fujimoto (2012), A long-term seafloor experiment using an acoustic ranging system: precise horizontal distance measurements toward detection of seafloor crustal deformation, Ocean Engineering, 51, 28–33.

Osada, Y., M. Kido, H. Fujimoto, and Y. Kaneda (2008), Development of a seafloor acoustic ranging system toward the seafloor cable network system, Ocean Engineering, 35, 1401–1405, doi:10.1016/j.oceaneng.2008.07.007.

Indeed, but these experiments were set-up in subduction zone environments. In 2014 we also deployed direct-path acoustic ranging beacons in another context in the Marmara Sea, which we did not refer to, given the limit in the number of references:

Sakic, P., Piété, H., Ballu, V., Royer, J.-Y., Kopp, H., Lange, D., et al. (2016). No significant steady state surface creep along the North Anatolian Fault offshore Istanbul: Results of 6 months of seafloor acoustic ranging. Geophysical Research Letters, 2016GL069600. https://doi.org/10.1002/2016GL069600

Lange, D., Kopp, H., Royer, J.-Y., Henry, P., Çakir, Z., Petersen, F., et al. (2019). Interseismic strain build-up on the submarine North Anatolian Fault offshore Istanbul. Nature Communications, 10(1), 3006. https://doi.org/10.1038/s41467-019-11016-z

Yamamoto, R., Kido, M., Ohta, Y., Takahashi, N., Yamamoto, Y., Pinar, A., et al. (2019). Seafloor geodesy revealed partial creep of the North Anatolian Fault submerged in the Sea of Marmara. *Geophysical Research Letters*, *46*(3), 1268–1275. https://doi.org/10.1029/2018GL080984

• Line 68: I think it's important to briefly mention how BPR drift is controlled, or what type of BPR this is (a commercial or custom instrument).

This is now detailed in the Methods section (which we believe is more appropriate than in the main text).

• Line 70: Fox (1999) might be a better reference than Fox (1993) here, because it refers to the same event as the Dziak and Embley references, and it has a more definitive signal.

Fox, C. G. (1999), In situ ground deformation measurements from the summit of Axial Volcano during the 1998 volcanic episode, Geophys. Res. Lett., 26(23), 3437–3440.

Indeed, the reference has been replaced.

• Line 75-76: This description is slightly confusing, because the Mw=4.92 event was not the first detected earthquake, it was the first in the GCMC catalog with a focal mechanism determination (as shown in Figure 2). The hydrophone array

detected some earthquakes a few minutes before this one. So probably just need to rephrase this slightly to acknowledge this.

We agree and have rephrased the text accordingly.

- Line 77: Looks like 17 hydroacoustic events in figure 2A (rather than 15).

We carefully recounted the events in Fig. 2A and checked our catalog, there are only 16 AUH events + 2 GCMT events in this first phase.

- Lines 81-82: Could also add Dziak and Fox (1999a) here.

Done.

- Lines 87-89: This step-wise co-seismic subsidence really reminds me of the caldera collapse sequence at Kilauea volcano, Hawaii, in 2018, described in Neal et al., 2019 (although obviously a different setting):

Neal, C. A., et al. (2019), The 2018 rift eruption and summit collapse of Kīlauea Volcano, Science, 363, 367–374, doi:10.1126/science.aav7046.

Referred to further down at line 217 (203 in the original ms).

- Line 119: This should be Fox (1999), rather than Fox (1993) ... but actually maybe that should just be omitted, because that paper is about vertical deformation, whereas this sentence is talking about horizontal displacements. Also, the Chadwick et al reference is maybe not a great analogy, because that horizontal ranging was not across a zone where a dike was intruded. Nevertheless, it is true that the changes measured were much smaller in that case.

Left as is.

- Line 125-126: Is the built-in pressure data shown somewhere?

We have added a panel (panel c) to Extended Data Figure 2 showing the raw pressure data from the BPR as well as the detided pressure data from acoustic transponder TP7 (requested by Reviewer 2 in his PDF annotations).

- Line 132: Nooner & Chadwick (2016) model co-eruption subsidence during the 2015 eruption at Axial Seamount. They do consider a penny-shaped crack sill model, but their preferred deformation model is not a sill. You might consider adding this reference, which does include various sill models for the same dataset, but also considering newer AUV repeat bathymetry data:

Slead, S., M. Wei, S. L. Nooner, W. W. Chadwick Jr., D. W. Caress, and J. W. Beeson (2024), Compartmentalization of Axial Seamount's magma reservoir inferred by analytical and numerical deformation modeling with realistic geometry, Journal of Geophysical Research: Solid Earth, 129(5), e2023JB028414, doi:10.1029/2023JB028414.

We have added this reference.

- Line 152: A couple of alternative references to consider rather than Chadwick et al., 1998:

Fox, C. G., W. W. Chadwick, Jr., and R. W. Embley (1992), Detection of changes in ridge-crest morphology using repeated multibeam sonar surveys, Journal of Geophysical Research: Solid Earth, 97, 11149–11162, doi:10.1029/92JB00601.

This was the first time repeat bathymetry was done on a MOR, but it was pre-GPS navigation, so we had to have extra steps to align the bathymetric grids. The Chadwick et al. 1998 paper also had to deal with pre-GPS navigated bathymetry, so is noisier than is possible now. A post-GPS example would be this one (related to the 2015 eruption at Axial Seamount):

Chadwick, W. W., Jr., B. P. Paduan, D. A. Clague, B. M. Dreyer, S. G. Merle, A. M. Bobbitt, D. W. Caress, B. Philip, D. S. Kelley, and S. L. Nooner (2016), Voluminous eruption from a zoned magma body after an increase in supply rate at Axial Seamount, Geophys. Res. Lett., 43, 12063–12070, doi:10.1002/2016GL071327.

You could just say “grids” instead of “Digital Terrain Models”.

These 2 references are indeed more pertinent.

- Lines 155-156: To me it looks like there are (maybe) two parallel lines of depth change (lava flows) in Figure 3 (also Extended Data Figure 6). I'm curious of the authors interpret this as due to eruption from two parallel dikes (which would be unusual), or alternatively that either the flows are all connected by thinner portions that are below the detection limit,

or perhaps that magma also intruded up along the normal fault at the western edge of the axial valley. I'd be curious to see the (noisier) raw difference grid; Figure 3 looks like it only shows depth differences above a 5 m threshold. Maybe that should be added as another panel in Extended Data Figure 6. The caption to Extended Data Figure 6 seems to suggest the authors favor the second of my alternative hypotheses above (magma also intruded up along the western fault). I think this interpretation should be added as a sentence or two in the main text (with appropriate acknowledgement of uncertainty) and not be buried in the caption of an Extended Data figure.

We agree that a single dyke is more likely than 2 dykes; this was our interpretation, which, as you pointed out, is better mentioned in the text (lines 158-159) than hidden in a figure caption.

• Line 163: Could add a reference to Chadwick et al. (2016) here, since the volume of the 2015 eruption at Axial Seamount about about 150 million m3.

Chadwick, W. W., Jr., B. P. Paduan, D. A. Clague, B. M. Dreyer, S. G. Merle, A. M. Bobbitt, D. W. Caress, B. Philip, D. S. Kelley, and S. L. Nooner (2016), Voluminous eruption from a zoned magma body after an increase in supply rate at Axial Seamount, Geophys. Res. Lett., 43, 12063–12070, doi:10.1002/2016GL071327.

Clague et al. (2017) has a slightly refined volume estimate for the same eruption:

Clague, D. A., J. B. Paduan, D. W. Caress, W. W. Chadwick Jr., M. L. Saout, B. Dreyer, and R. Portner (2017), High-resolution AUV mapping and targeted ROV observations of three historical lava flows at Axial Seamount, Oceanography, 30(4), 82–99, doi:10.5670/oceanog.2017.426.

Done.

• Line 167: Need to define "H-wave" the first time you use it.

Done (in text and Methods).

• Line 168: Lava-water interaction signals were also described in these references from the 2015 Axial Seamount eruption:

Wilcock, W. S. D., M. Tolstoy, F. Waldhauser, C. Garcia, Y. J. Tan, D. R. Bohnenstiehl, J. Caplan-Auerbach, R. P. Dziak, A. F. Arnulf, and M. E. Mann (2016), Seismic constraints on caldera dynamics from the 2015 Axial Seamount eruption, Science, 354(6318), 1395–1399, doi:10.1126/science.aah5563.

Le Saout, M., D. R. Bohnenstiehl, J. B. Paduan, and D. A. Clague (2020), Quantification of eruption dynamics on the north rift at Axial Seamount, Juan de Fuca Ridge, Geochem. Geophys. Geosyst., 21, e2020GC009136, doi:10.1029/2020GC009136.

Indeed, we were aware of these observations, but the hydrophones were at a much closer range (few km). Nevertheless, we added the references (lines 167 and 176 in the revised manuscript).

• Line 171: This is also the interpretation from the 2015 Axial eruption - that the hydroacoustic impulsive events mark the duration of lava extrusion. (Wilcock et al., 2016; Le Saout et al., 2020). Can the impulsive hydroacoustic events be located? If so, do they cluster where the depth changes occur? What do their locations in space and time tell us about the emplacement of the lava flows [like Le Saout et al. (2020)]? Will this be part of a future study?

Yes. As outlined further below (comment on Extended Data Figure 2), since our initial submission, we automatically processed the hydroacoustic data searching for these impulsive events and detected more than 4000, of which, as an initial check, we only retained those we could trilaterate (i.e. recorded by at least 3 hydrophones). They span obliquely relative the main extrusion sites, given the array geometry. Further analyses are under way.

• Line 203: Maybe another place to point out the similarity to Kilauea's 2018 collapse (Neal et al., 2019)?

Done (now line 216).

• Line 472: I added this sentence to the end of this paragraph: “ We may be able to reduce the uncertainty in the ranging measurements further after all the instruments and their recorded (temperature) data are recovered at the end of the experiment.” Is this true?

We are counting on it in our future analyses. First, sound-speed will be better assessed when we have the temperature and pressure at both ends of each baseline. Second, with our network of baselines, knowing the changes in the tripod inclination (i.e. acoustic head displacements), we may assess their orientation from multiple

baselines originating from the same station: a tilt should be seen as lengthening on some direction and shortening in the opposite direction. We are currently trying to implement a method on synthetic data.

• Figure 1B: There is a lot of information in this figure, which is great, but I worry that it will not be legible if it is reduced any in size when published (the font size showing the earthquake magnitudes is already very small, for example).

The size of all texts has been enlarged.

• Figure 2A: Change label here from “BPR” to “BPR location”.

Done + text enlargements.

• Figure 3: Maybe make magma lens a different color (light red?), to avoid confusion because the lava flows are grey? If so, then make the dashed line representing the model dike be a darker red, so it stands out. Also make the labels “Dike” and “Magma lens” the appropriate colors. Add black lines to the tops of the inset plots, so they are completely enclosed and separated from the underlying plot.

Done, it does make the figure more legible. Colors were changed to match those in Figure 3.

• Figure 4: Consider changing the label “Baseline changes” to “Horizontal range baseline changes”, just to clarify. If you do that, adjust the wording in the figure caption too. In the caption, I assume you aren’t really “summing up” the horizontal and vertical displacements, but rather using them to calculate a range change or slope distance, right? This should be rephrased to clarify.

Baselines changes are really baselines changes, not only horizontal. Since TP7 subsided by more than 4m, it contributed to horizontal + vertical baseline changes. This is the reason why we considered ΔL (see Methods).

• Extended Data Figure 2: What happens after May 2? The number of impulsive events has not leveled off by then. How long does it keep increasing? Or when do the impulsive events stop?

Good question. When we first submitted the paper, we were not done with all hydroacoustic analyses. In this revised version, we have added a plot showing automatically detected impulsive events (panel b in Extended Data Fig. 2), which shows that impulsive events continue in 3 discrete bursts through May 12, 2024, with a small additional burst occurring mid-June. We thus believe that the bulk of the lava effusion was over by May 12, which we use to provide an order of magnitude estimate of the mean extrusion rate (116 m^3/s line 172).

• Extended Data Figure 4: I would change the label on the figure from "drainage" to "sill"

Done.

• Extended Data Figure 5: Also explain what the brown histograms are in the caption.

Done.

**********************************END***********************************

Changes in the figures:

Figure 1: All magnitudes and event numbers were enlarged, as suggested.

Referee #2: There is a lot of information in this figure, which is great, but I worry that it will not be legible if it is reduced any in size when published (the font size showing the earthquake magnitudes is already very small, for example).

Figure 2 (referee #2):

BPR location added, text, magnitudes and stars enlarged, Mw in black, mb in blue as in Fig 1b

Figure 3 (referee #2):

Referee #2: Maybe make magma lens a different color (light red?), to avoid confusion because the lava flows are grey? Add black lines to the tops of the inset plots, so they are completely enclosed and separated from the underlying plot.

Colors for dyke and magma lens (sill) changed to match those in model of Figure 4 + framed insets.

Figure 4 (referee #2):

Referee #2: I assume you aren't really "summing up" the horizontal and vertical displacements, but rather using them to calculate a range change or slope distance, right? This should be rephrased to clarify.

Consider changing the label "Baseline changes" to "Horizontal range baseline changes", just to clarify. If you do that, adjust the wording in the figure caption too.

Reply: Label left unchanged, because they are "overall" baseline changes (horizontal+vertical, see Methods), as now stated in the caption. Panels are now labelled a, b

Figure 5:

Entirely redone as its caption to address Reviewer #1 main comment and to illustrate the revised text.

Extended Data Table 1:

All Mw magnitudes were rounded up to the 1st decimal (reviewer #1).

Extended Data Figure 1:

Updated according to the revised calculations (reviewer #1 comment) (updated in the Methods).
+ Mw magnitudes rounded up to 1 decimal

Extended Data Figure 2:

Referee #2: What happens after May 2? The number of impulsive events has not leveled off by then. How long does it keep increasing? Or when do the impulsive events stop?

Updated with 3 additional panels (b,c,d) to address these questions. Hydroacoustic data were further processed and impulsive event statistics/curves updated accordingly (panels a and b).

Extended Data fig. 3 and former fig. 8 (baselines) have been merged into a single figure with 2 panels, for more conciseness.

Extended Data Figure 4:

Referee #2: Drainage changed to sill

Extended Data Figure 5:

Panel c updated to better illustrate the revised text.

Extended Data Figure 6:

Lower panel (lava thickness > 2m) added to address the query from reviewer #2.
Caption changed accordingly, including the scenario for the source of lavas, described in the revised text

Extended Data Figure 8:

Referee #2: ADD colour scale for lavas [added + enlarged numbers]

Ref: MS 2025-11-29673 - ***Anatomy of a seafloor spreading event captured by in-situ seismo-geodesy***

Response to the referees' comments, May 6, 2026.

Referee #1:

This manuscript represents a revised version of a paper I had previously reviewed. I greatly appreciate that most of my comments were appreciated and the criticism acknowledged and hence the manuscript greatly advanced – especially the discussion of the released seismic budget and its relationship to the adjacent transform faults. There are only a very few and very minor points – which I believe can also handled during final editing of the manuscript and hence after it acceptance.

Line 90: GCMT centroid depth is always given below sea level – though the authors state it is below seafloor. However, I checked the value reported in the online catalogue and the reported depth agrees which the online catalogue, hence are below sea level. Please correct.

We have checked the three events in question in the GCMT catalogue. The catalogue indeed reports depth below sea-level that range between: 13.6 km (event **202404262023A**) and 18.1 km (event **202404262009A**). The third event has a depth of 14.4 km (event **202404262054A**).

For consistency with the preceding sentence, we chose to report depths below seafloor and thus subtracted 2 km water depth (as explained in our initial rebuttal letter), yielding a range of 11.6 to 16.1 km, which we rounded to 12–16 km below seafloor. We have thus triple-checked these values and stand by them.

Lines 133-145: Paragraph on dislocation modelling – the paragraph is largely written for a community dealing with modelling of similar types of problems. Thus, I do not know how many readers will understand it. For example, there might be a large number of reader who may not understand well what compaction of a sill by 10-20 m (line 136) suggests. Further, give the range for the opening (line 137) – you just say meters; however, just 1-2 m or tens of metres? The same holds for the slip (line 137, too). Please give the range – 1-2 m or more? The careful reader can deduce those from the extended figures – but it should be clear just by reading the text.

We have added (l. 137-139) specific ranges of numbers for the sill compaction, dyke opening, depth and fault slip so that the reader does not need to look for them in **Extended Data Figure 5**. We calculated those ranges based on the 16th and 84th percentiles of our model distributions (equivalent to ±1 standard deviation if the distribution was purely Gaussian), as it is now mentioned in the Methods section (l. 433-435). This yields:

- sill compaction 12–18 m
- dyke opening 1.0–3.3 m
- shallowest depth reached by dyke 83–377 m
- fault slip 0.6–3.1 m
- total extension 2.1–4.0 m

To clarify the meaning of "*sill compaction*", we have rephrased it as "*a sill collapsing by 12-18 m*".

Also, to make it clearer to the reader, we added some small arrows in **Figure 4b** to show what type of motion is expected along the fault, dyke, and sill, and also added a sentence to explain what they are in the legend.

Line 139-140: Figure 4 does not show any sill that is 2500 m (i.e, 2.5 km!) wide. Actually, a 2500 m wide sill would build nearly half of the crust in just one event; rather unlikely. Please correct or describe the process more clearly.

The blue segment shown in **Figure 4b** matches the standard definition of a sill: a tabular, horizontal sheet intrusion. This terminology is commonly used in the mid-ocean ridge literature to describe the horizontal, lens-shaped and elongated magma reservoirs that are seismically imaged at shallow depths beneath the ridge axis (e.g., Sinton & Detrick, 1992). We believe the reviewer may be confused by our use of the word "width", intended to mean "horizontal cross-axis extent of 2500 m" (again typical for mid-ocean ridges). Perhaps the reviewer understood "width" (measured horizontally) as a "thickness" (measured vertically) of 2500 m, which would indeed

build an unrealistic amount of crust at once! We have no constraint on the actual thickness of the sill, but our geodetic measurements and models suggest that this thickness was reduced by 12-18 m during the eruption. This is what we call "*compaction*", or "*deflation*" in other parts of the manuscript. We have attempted to further clarify the terminology by adding the following sentence in the caption of **Extended Data Fig.4** (l. 613-614): "*Compaction of a magma lens below TP7 (i.e. deflation of a reservoir due to magma withdrawal) ...*".

New Figure 5: the left-hand side of the plot is "half empty" (Fig 5a). Why not cut the vertical axis at 1000 PJ/km?

We agree that panel a contains a lot of empty space, but, unfortunately, panels a and b are at the same scale in equivalent units. The vertical axes between panels must be aligned so that the dashed red line in panel a "arrives" near the mode of the pink distribution in panel b (i.e. moment of 21.82 PJ/km = 315 m^3/m of horizontal potency or 900 PJ/km ~ 13,000 m^3/m). Cutting the y-axis at 1,000 PJ/km would hide a significant portion of the spread of the measured extension (that goes up to 23×10^3 m^2 or 1,600 PJ/km).

Great experiment! Ingo Grevemeyer

****

Referee #2:

Review of revised manuscript by William Chadwick

I find that the authors have addressed all my earlier comments, concerns, and suggested edits. The only remaining issue I noticed was that in the caption to Figure 3, it says "(light red rectangle)" when referring to the deflating magma lens in the figure, but it should say "(purple rectangle)", because the authors changed the color of the rectangle during revision. Otherwise, I think the paper is ready to publish. I note that the authors have made some very valuable additions to the Extended Data Figures, based on my previous comments. This is a very remarkable and exciting paper!

We have changed the legend of **Figure 3** accordingly (l. 539).
